# Peptide-MHC–targeted retroviruses enable in vivo expansion and gene delivery to tumor-specific T cells

**DOI:** 10.1126/sciadv.adv2331

**Published:** 2025-11-07

**Authors:** Ellen J. K. Xu, Blake E. Smith, Winiffer D. Conce Alberto, Michael J. Walsh, Birkley Lim, Megan T. Hoffman, Li Qiang, Ariana Barreiro, Emma N. Finburgh, Jiayi Dong, Andrea Garmilla, Qingyang Henry Zhao, Caleb R. Perez, Stephanie A. Gaglione, Connor S. Dobson, Michael Dougan, Stephanie K. Dougan, Michael E. Birnbaum

**Affiliations:** ^1^ Department of Biological Engineering, Massachusetts Institute of Technology, Cambridge, MA, USA.; ^2^ Koch Institute for Integrative Cancer Research, Cambridge, MA, USA.; ^3^ Singapore-MIT Alliance for Research and Technology Centre, Singapore, Singapore.; ^4^ Department of Immunology, Harvard Medical School, Boston, MA, USA.; ^5^ Department of Cancer Immunology and Virology, Dana-Farber Cancer Institute, Boston, MA, USA.; ^6^ Division of Gastroenterology, Massachusetts General Hospital, Boston, MA, USA.; ^7^ Department of Chemical Engineering, Massachusetts Institute of Technology, Cambridge, MA, USA.; ^8^ Department of Medicine, Harvard Medical School, Boston, MA, USA.; ^9^ Ragon Institute of MGH, MIT, and Harvard, Cambridge, MA, USA.

## Abstract

Tumor-infiltrating lymphocyte (TIL) therapy has demonstrated that endogenous T cells can be harnessed to initiate effective antitumor responses. Despite clinical promise, current TIL production protocols involve weeks-long ex vivo expansions that can affect treatment efficacy. Therefore, additional tools are needed to engineer TILs to have increased potency while mitigating manufacturing challenges. Here, we present a strategy for pseudotyping retroviruses with peptide–major histocompatibility complexes (pMHCs) for antigen-specific gene delivery to CD8 T cells and validate therapeutic impact in immunocompetent mouse models. We demonstrate that pMHC-targeted viruses specifically deliver function-enhancing cargos while simultaneously activating and expanding antitumor T cells. This targeting precision enables in vivo engineering of tumor-specific T cells, resulting in improved overall survival in B16F10-bearing mice. Together, we have established that pMHC-targeted viruses are efficient vectors for reprogramming and expanding tumor-specific T cells directly in vivo, with the potential to substantially streamline engineered cell therapy production.

## Teaser

Viral vectors displaying peptide-MHC are versatile tools for activating and reprogramming antitumor CD8 T cells directly in vivo.

## INTRODUCTION

Adoptive cell therapy is a promising cancer treatment modality that uses expanded or genetically modified T cells to recognize and potently kill tumor cells. Tumor-infiltrating lymphocyte (TIL) therapy was approved by the Food and Drug Administration in 2024 for the treatment of advanced melanoma, becoming the first cell therapy approved for solid tumors (*1*). TIL therapies are produced by isolating autologous T cells from tumor resections and extensively expanding the cells ex vivo before reinfusing them into the patient. Early exploration of TIL therapy highlighted its ability to induce long-lasting remission in patients with metastatic melanoma (*2*, *3*), and since then, varying frequencies of TILs have been detected in a range of cancers (*4*–*7*), underscoring the potential benefit of using these cells as a treatment for multiple cancer types.

The phenotype and quality of TIL products have been demonstrated to substantially influence clinical outcomes. Before reinfusion, TILs often exhibit exhausted phenotypes due to their previous intratumoral antigen exposure and the prolonged ex vivo expansion protocol, impeding them from mounting an aggressive and durable antitumor response (*8*). To mitigate this, additional engineering strategies to improve cell potency could be incorporated into production protocols with the goals of delivering immunostimulatory cargos (*9*, *10*) and diminishing immunosuppressive signals (*11*–*13*). However, current methods to introduce these modifications would apply them to all T cells present in the TIL expansion, increasing potential toxicities resulting from engineered bystander T cells while further increasing manufacturing complexity. Evidence from other engineered cell therapies suggests that the persistence and efficacy of adoptively transferred cells diminishes as the interval between T cell isolation and reinfusion increases (*14*, *15*), reinforcing the need to minimize production time to increase treatment success. Additional strategies are therefore desirable to revitalize and generate a more effective antitumor response before engineered TIL therapies can be widely translated to the clinic.

Developing gene delivery vectors to modify tumor-specific T cells directly in vivo could mitigate many of these challenges involving ex vivo TIL manufacturing and potency. Ongoing efforts to generate viral vectors for in vivo gene delivery are focused on limiting viral tropism to specific T cell lineage markers such as CD3, CD8, CD4, CD62L, and CD5 (*16*–*23*). While these strategies may be appropriate for the generation of chimeric antigen receptor (CAR) T cells, since they will modify broad subsets of T cells, they are less appropriate for antigen-specific transduction, which could enhance the efficacy of TIL therapies. Recently, targeted gene delivery to antigen-specific T cells has been demonstrated, relying on display of peptide–major histocompatibility complex (pMHC) to be recognized by a T cell clone’s unique T cell receptor (TCR) (*24*–*26*). The development of lipid nanoparticles targeted by pMHC have demonstrated that in vivo antigen-specific delivery of mRNA-encoded cargos is possible (*25*), yet longer term transgene expression will likely be required to ensure robust antitumor responses. While there is some precedent that functional cargos can be delivered using lentiviral vectors via TCR-pMHC interactions in vitro (*26*), no studies have evaluated the efficacy of viruses displaying pMHC (pMHC-targeted viruses) as antitumor immunotherapeutic agents.

Here, we demonstrate that pMHC-targeted viruses can be used to efficiently reprogram antitumor T cell populations, delivering a therapeutic cargo that generates an effective antitumor response in vivo. First, we developed a pMHC-targeted gammaretrovirus pseudotyping strategy to facilitate efficient gene delivery to murine antigen-specific T cells in polyclonal populations, enabling evaluation of treatment efficacy in immunocompetent models. We demonstrated our approach using a tethered version of the cytokine interleukin-12 (IL-12) as a test cargo, given its potent immunomodulatory effects but dose-limiting toxicities when delivered systemically (*27*, *28*). Transducing antigen-specific T cells in vitro, we observed activation and expansion of target T cells that were able to extend survival in a B16F10 melanoma model. Last, we determined that pMHC-targeted viruses delivering tethered IL-12 could be infused directly in vivo to generate long-lived, memory T cells that extend tumor control. Together, we demonstrate that pMHC-targeted viruses are safe and effective vehicles for in vivo gene delivery to antitumor T cells, with the potential to customize these vectors for a wide range of antigen-specific T cell modifications.

## RESULTS

### pMHC-pseudotyped viruses efficiently activate, expand, and transduce murine CD8 T cells

Our laboratory and others have demonstrated that lentiviruses can be pseudotyped via coexpression of pMHC and a receptor-blinded version of the vesicular stomatitis glycoprotein (termed “VSVGmut”) that can serve as a fusogen to enable antigen-specific transduction of human T cells (*24*, *26*, *29*). To evaluate the potential of pMHC-targeted viruses as gene delivery vectors, we first established a murine model to study the full scope of function of virally engineered antigen-specific T cells. Rather than rely on xenograft models, which would restrict evaluation of cargos to those that could function without a complete immune system, we chose to adapt pMHC-targeted vectors for use in syngeneic models.

**Fig. 1.**
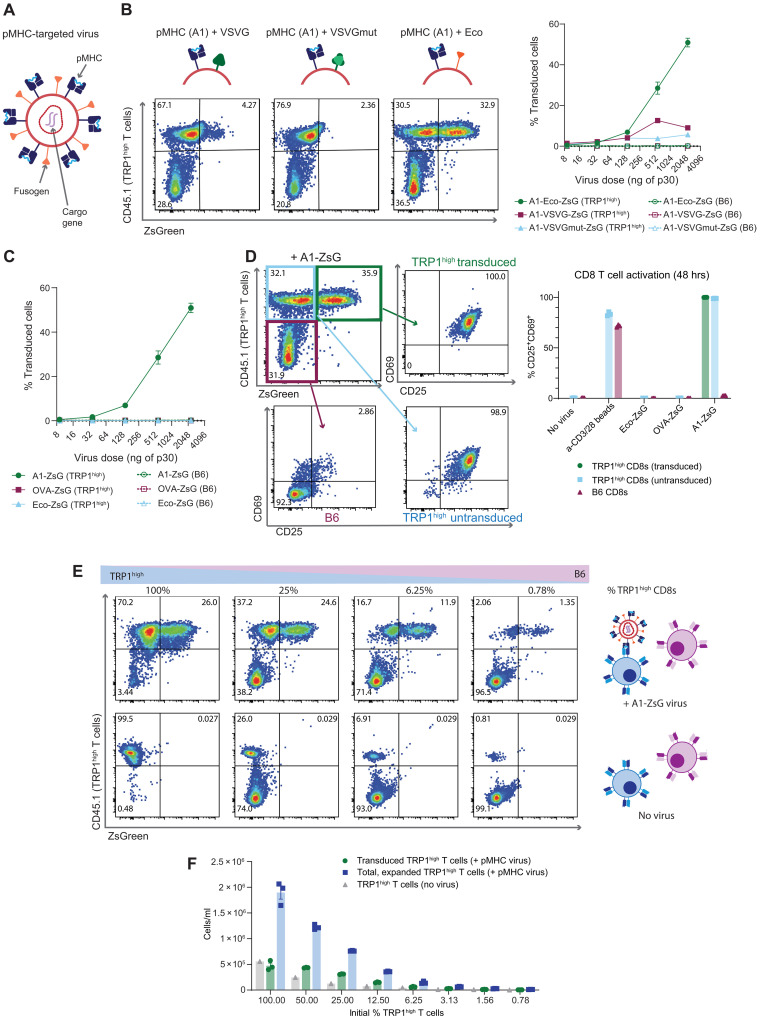
Viral vectors coexpressing pMHC and the MuLV ecotropic envelope selectively activate, expand, and transduce antigen-specific murine CD8 T cells. (**A**) Schematic of pMHC-targeted gammaretrovirus detailing the coexpression of the targeting (pMHC) and fusogen molecules on the viral membrane and the therapeutic cargo for delivery packaged in the viral genome. (**B**) Different pMHC + fusogen combinations were tested to determine the most efficient pseudotype. Viruses delivering ZsGreen were added to a mixture of 50% on-target (TRP1^high^) and 50% off-target (B6) CD8 T cells. Forty-eight hours later, percent transduction was calculated of the on-target or off-target population, with representative flow plots shown. Values plotted are means ± SEM. (**C**) The same experimental protocol as (B) using pMHC-targeted viruses displaying an on-target pMHC (A1-ZsG), off-target pMHC (OVA-ZsG), or no pMHC (Eco-ZsG). Data shown are means ± SEM. (**D**) Representative plot from (B) highlighting expression of activation markers CD25 and CD69 in TRP1^high^ transduced, TRP1^high^ untransduced, and off-target B6 CD8 T cells 48 hours after addition of A1-Eco-ZsGreen virus (2310 ng of p30 dose). Summary plot showing percent CD25^+^CD69^+^ after adding pMHC-targeted viruses or anti-CD3/CD28 beads. Individual values shown with bars plotting means ± SEM. (**E**) Representative flow plots depicting different starting frequencies of TRP1^high^ T cells mixed with B6 CD8 T cells (bottom row) and the resultant expansion and transduction (top row) 48 hours after adding A1-ZsG virus. The same dose of A1-ZsG virus (924 ng of p30) was added to all populations. (**F**) Absolute cell counts from experiment in (E) shown as individual values with bars plotting means ± SEM.

To demonstrate our approach, we used TRP1^high^ TCR transnuclear mice as a source of CD8 T cells that recognize a well-described melanoma antigen, tyrosinase-related protein 1 (TRP1), with physiological pMHC:TCR affinity (*30*). On the basis of previous studies using the TRP1^high^ system, we selected the strong agonist peptide mimotope of the TRP1 epitope, AAPDLGYM, presented by the murine class I MHC H2-D^b^ in the format of a single-chain pMHC trimer (termed “A1”), as an initial targeting molecule for proof-of-concept experiments (*31*–*33*). Given previously reported limitations of using lentiviruses to efficiently infect murine T cells (*34*), we adapted our targeting approach for use with murine leukemia gammaretrovirus (MuLV) as the viral vector for codisplay of pMHC and fusogen (Fig. 1A).

We hypothesized that pMHC-displaying viral particles would selectively activate antigen-specific target cells in a population of naïve T cells. Therefore, we evaluated the efficiency of A1-targeted viruses displaying unmutated VSVG, VSVGmut, or the ecotropic envelope protein (Eco) derived from the Moloney murine leukemia virus, a commonly implemented gammaretroviral vector for gene delivery to preactivated murine CD8 T cells (*35*–*38*). Targeted gammaretroviruses delivering a fluorescent cargo (ZsGreen), each displaying A1 and a fusogen, were used to transduce mixtures of freshly isolated on-target (TRP1^high^) and off-target, polyclonal wild-type C57BL/6J (B6) murine CD8 T cells. After 2 days, the highest on-target transduction was mediated by viruses coexpressing A1 with the ecotropic envelope fusogen (A1-ZsG) (Fig. 1B). Viruses targeted with a noncognate pMHC (displaying an ovalbumin-derived peptide) or Eco alone did not mediate transduction in unactivated cells (Fig. 1C). However, when added to preactivated cells, the untargeted virus displaying only Eco (Eco-ZsG) was able to induce robust transduction of both TRP1^high^ and B6 CD8 T cells, validating that this parental virus was functional and not antigen specific (fig. S1). H2-D^b^-targeted viruses displaying a nonagonist TRP1 peptide TAPDNLGSM (*33*) (S8-ZsG) or an off-target influenza A antigen ASNENMETM (*39*) (NP-ZsG) were unable to transduce or activate TRP1^high^ T cells at all viral doses tested (fig. S2), further underscoring that the specificity of pMHC-targeted viruses is driven by TCR recognition of the cognate peptide presented by the displayed pMHC single-chain trimer.

We observed specific activation of on-target TRP1^high^ T cells when A1-targeted viruses were added to the CD8 T cell mixture, whereas off-target cells in the same well were not activated (Fig. 1D). Addition of untargeted Eco-ZsG or an off-target pMHC-ZsG virus did not result in similar levels of CD69 or CD25 expression in TRP1^high^ CD8 T cells compared to addition of A1-ZsG. Even in TRP1^high^ T cells that were not transduced to express ZsGreen, but were exposed to A1-ZsG virus, we observed similar proportions of CD25^+^CD69^+^ CD8 T cells compared to transduced TRP1^high^ T cells, indicating that pMHC-displaying viruses are potent activators of antitumor T cells.

We next evaluated the efficiency of A1-ZsG viruses at selectively transducing on-target cells at lower initial frequencies. In patient samples, the endogenous frequency of antitumor, cytotoxic T lymphocytes ranges from ~1% for MART-1–specific T cells to less than 0.01% for WT-1, MAGE family, and NYESO-1–specific T cells (*40*). To simulate this, we diluted TRP1^high^ CD8 T cells into B6 CD8 T cells at different ratios and found that A1-ZsG viruses were able to transduce on-target cells at all starting frequencies tested (Fig. 1E). Moreover, these pMHC-targeted viruses were able to expand rare, cognate T cells (Fig. 1F), suggesting that pMHC-targeted viruses could be used to deliver therapeutic cargos to enhance antitumor T cell function with the additional benefit of simultaneously expanding the overall number of antitumor, effector T cells.

**Fig. 2.**
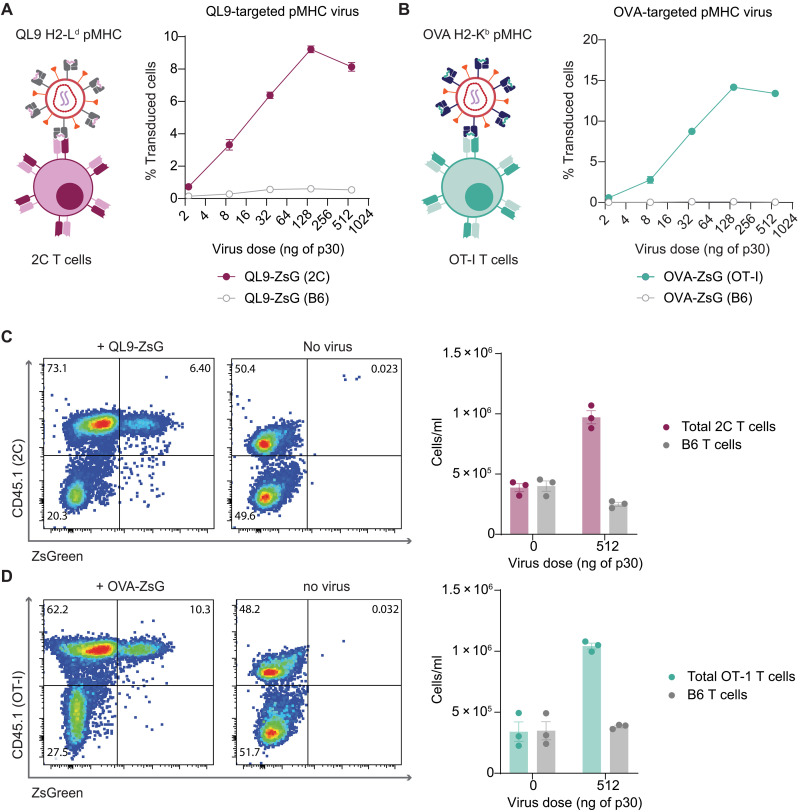
pMHC-targeted viruses are versatile tools for gene delivery to antigen-specific T cells in a variety of model systems. (**A** and **B**) pMHC-targeted gammaretroviruses can be retargeted to different antigen-specific T cell populations by changing the single-chain trimer pMHC that is coexpressed with the ecotropic envelope glycoprotein. Two different ZsGreen viruses were generated, displaying a QL9 or OVA peptide in different MHC alleles to target 2C or OT-1 T cells, respectively. Transduction as measured after 48 hours post–virus addition is shown for QL9-ZsG and OVA-ZsG viruses added to a 1:1 ratio of on-target (cognate transgenic TCR T cells) and off-target cells (B6 CD8 T cells). Data representative of two separate experiments and depicted as means ± SEM. Percent transduced cells shown on the *y* axis was calculated within the on-target or off-target populations. (**C** and **D**) Representative flow plots from (A) and (B) where on-target virus (dosed at 512 ng of p30) was added to a 1:1 mixture of on- and off-target cells (left) compared to a control well (right) where no virus was added. Absolute cell counts are plotted for total on-target cells (magenta or teal bars) and off-target cells (gray bars) at indicated doses of virus depicted as individual values with means ± SEM.

To validate the generality of our pMHC-targeting approach, we next demonstrated that pMHC-targeted viral vectors can be readily adapted for other antigen targets. To this end, we generated single-chain trimer versions of H2-K^b^ presenting the ovalbumin-derived model antigen SIINFEKL (OVA) (*41*), and H2-L^d^–presenting QLSPFPFDL (QL9), an agonist for the 2C TCR model system (*42*, *43*). Individually pseudotyping viruses with each of these pMHC plus Eco, we observed that the vectors maintained high specificity for their cognate TCR compared to off-target B6 CD8 T cells (Fig. 2, A and B). Furthermore, in each of these additional systems, pMHC-targeted viruses both expanded and transduced on-target T cells (Fig. 2, C and D). Together, we have developed a modular pMHC-based pseudotyping strategy that enables specific activation, expansion, and transduction of murine antigen-specific T cells from rare, polyclonal populations in vitro.

### A1-targeted viruses can deliver a functional, tethered mIL-12 cargo

**Fig. 3.**
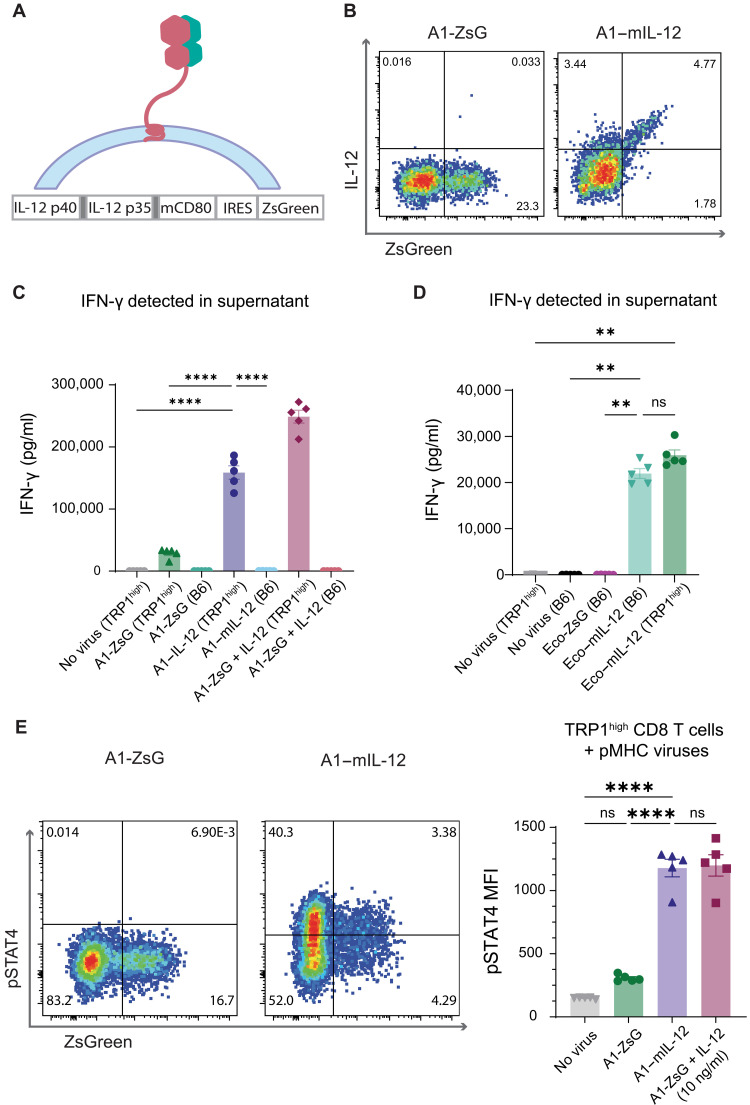
pMHC-targeted retroviruses enable delivery of a functional cargo, tethered IL-12, to tumor-specific T cells. (**A**) Construct design of tethered IL-12, consisting of a single-chain IL-12 attached via a flexible linker to the transmembrane domain of murine CD80. Shaded gray boxes represent flexible linkers. (**B**) Representative flow plots of transduction achieved with A1-ZsG or A1–mIL-12 viruses added to TRP1^high^ CD8 T cells after 48 hours. Single experiment from *n* = 3 biological replicates. (**C**) IFN-γ secretion in supernatants from cultures of isolated TRP1^high^ CD8 T cells 48 hours after transduction with A1-targeted viruses. Virus doses were standardized to 888.9 ng of p30 per 150,000 cells. In the A1-ZsG + IL-12 condition, cells were resuspended in media supplemented with mIL-12 (10 ng/ml) and transduced with A1-ZsG. Individual values with means ± SEM plotted. *P* values were calculated by Bonferroni-corrected one-way analysis of variance (ANOVA). ****P < 0.0001. (**D**) IFN-γ secretion in supernatants from cultures of preactivated cells transduced with Eco-targeted viruses. Supernatants were collected 48 hours after transduction. Virus doses were matched to those used in (C). Individual values with means ± SEM shown. Significance was determined using Bonferroni-corrected one-way ANOVA. **P < 0.01. (**E**) TRP1^high^ CD8 T cells were isolated and transduced with A1-ZsG or A1–mIL-12 virus. Forty-eight hours after transduction, cells were fixed, permeabilized, and stained with anti-pSTAT4 antibody before analysis via flow cytometry. Cells were transduced with A1-ZsG virus and cultured in media with IL-12 (10 ng/ml) as a positive control. Individual values with bars indicating means ± SEM shown. *P* values were calculated by Bonferroni-corrected one-way ANOVA. ****P < 0.0001.

Many genetic cargos have been previously reported to enhance the potency, trafficking, or safety of adoptively transferred engineered T cell therapies (*44*). We hypothesized that our pMHC-targeted viruses would be particularly useful for delivery of therapeutic cargos that are limited by systemic toxicities. IL-12 has been recognized to enhance antitumor immunity but has faced challenges in its clinical implementation given its potent, dose-limiting side effects (*45*). We selected a tethered murine IL-12 (mIL-12) construct as a proof-of-concept cargo for genetic manipulation of antigen-specific T cells whose efficacy had previously been validated in conjunction with CAR-T cell therapy (Fig. 3A) (*46*). We confirmed that mIL-12 is detectable on the surface of cells transduced with targeted A1–mIL-12 viruses using an anti–mIL-12 antibody (Fig. 3B). When A1 viruses encoding mIL-12 were added to a mixed population of on- and off-target CD8 T cells, A1-targeted vectors maintained specificity for TRP1^high^ T cells as demonstrated by selective transduction of on-target cells (fig. S3). IL-12 signaling drives interferon-γ (IFN-γ) secretion, which was detectable in the supernatants of all conditions transduced with mIL-12 viruses (Fig. 3, C and D, and fig. S4). These results further highlight the specificity of A1-targeted viruses, as IFN-γ secretion is significantly elevated when A1–mIL-12 virus was added to TRP1^high^ T cells but not to polyclonal B6 T cells (Fig. 3C). In contrast, untargeted Eco–mIL-12 virus transduced and elicited IFN-γ secretion in both TRP1^high^ and B6 T cells (Fig. 3D and fig. S4B). Increased pSTAT4 signaling was also observed in TRP1^high^ cells transduced with A1–mIL-12 virus compared to A1-ZsG virus, demonstrating that the mIL-12 construct initiates expected downstream signaling cascades in transduced T cells (Fig. 3E). Elevated pSTAT4 levels were also detected in untransduced TRP1^high^ T cells, indicating that the tethered cytokine is able to mediate effects in trans. Together, these data demonstrate that pMHC-targeted viruses are able to specifically deliver a functional cargo in vitro.

### Antitumor T cells transduced with tethered IL-12 ex vivo extend survival in B16F10 tumor–bearing mice

**Fig. 4.**
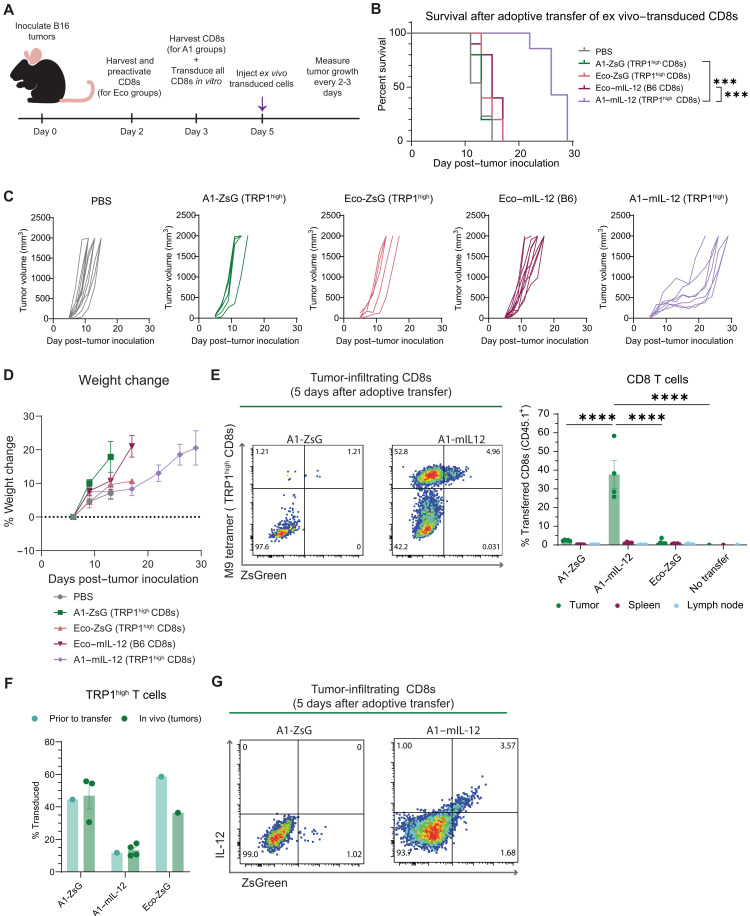
Adoptive transfer of TRP1^high^ T cells transduced by pMHC-targeted viruses delivering tethered IL-12 extends survival of B16F10 tumor–bearing mice. (**A**) Experimental design with additional details described in Materials and Methods. Donor CD8 T cells were isolated and transduced ex vivo with virus at a dose of 7.90 × 10^3^ ng of p30/1M cells. (**B**) Kaplan-Meier curve for overall survival. Shown here is one of three total experiments conducted. *P* values were determined using a Bonferroni-corrected log-rank (Mantel-Cox) test. ***P < 0.001. (**C**) Individual tumor growth curves from mice in (B). (**D**) Mouse weights were monitored every 2 to 4 days, and change in weight was normalized to 1 day after cell injection. Values are means ± SEM. (**E**) Representative plots of tumor-infiltrating CD8 T cells 5 days after adoptive transfer, gated on live CD8 T cells. For all groups, a dose of 1.08 × 10^4^ ng of p30/1M cells of virus was used to transduce cells. Summary plot of transferred cells detected in each tissue with individual values and means ± SEM depicted. *P* values were calculated by Bonferroni-corrected two-way ANOVA. ****P < 0.0001. (**F**) Percent transduction of total TRP1^high^ T cells in tumor samples or ex vivo before infusion. Samples where number of transferred cells detected was less than 10 were excluded from analysis. Individual values with means ± SEM plotted. (**G**) Representative panels of IL-12 and ZsGreen expression in tumor-infiltrating CD8 T cells gated on live CD8s of samples in (D). Each plot represents one mouse.

Having demonstrated the ability of pMHC-targeted viruses to deliver immunostimulatory cargos in vitro, we next explored how these transduced cells function in a therapeutic context. We selected B16F10 melanoma as an aggressive tumor model system that endogenously expresses the TRP1 antigen (*47*, *48*). After inoculating B16F10 tumor cells subcutaneously in B6 mice, we harvested donor CD8 T cells from transgenic TRP1^high^ mice and transduced the cells ex vivo with either A1-ZsG, A1–mIL-12, or untargeted Eco-ZsG (Fig. 4A). As an additional control, we transduced activated B6 CD8 T cells with Eco–mIL-12 virus to determine whether delivery of IL-12 to polyclonal T cells would be sufficient for therapeutic efficacy. After two days in vitro, the cell product from each group was assessed for baseline transduction and activation state before adoptive transfer (fig. S5).

Consistent with previous reports, TRP1^high^ T cells alone were not sufficient to control B16F10 tumors (*30*, *32*, *33*, *49*). However, mice that received TRP1^high^ T cells transduced with A1–mIL-12 virus were able to maintain smaller tumors at early time points, ultimately experiencing a significant survival benefit (Fig. 4, B and C). In contrast, delivery of tethered IL-12 to polyclonal CD8 T cells in the Eco–mIL-12 group did not lead to the same prolonged survival, indicating that tethered IL-12 must be specifically delivered to antitumor T cells for greatest therapeutic efficacy. While previous work has demonstrated that injections of soluble IL-12, even if given intratumorally, causes drastic initial weight loss in mice (*50*), no weight loss or other evidence of toxicity was observed in mice treated with TRP1^high^ T cells transduced with tethered mIL-12 via A1-targeted viruses (Fig. 4D).

Given the improvement in overall survival mediated by the transfer of A1–mIL-12 transduced cells, we next validated whether transduced cells could traffic to and persist in tumors. Following the same adoptive transfer protocol, TRP1^high^ T cells were transduced ex vivo and transferred into B16F10 tumor–bearing mice. Five days after transfer, tumors, spleens, and tumor-draining lymph nodes were harvested for analysis of infiltrating transferred and transduced cells. Across the tissues sampled, we detected an increased percentage of adoptively transferred CD8 T cells in tumors from the A1–mIL-12–transduced group compared to other experimental groups and in comparison to other tissues within the A1–mIL-12 treatment group (Fig. 4E). These results indicate that TRP1^high^ T cells are able to preferentially and efficiently traffic to tumors. In all virus-transduced groups, the fraction of transferred cells detected that were transduced (ZsGreen^+^) mirrored the baseline frequency of ZsGreen in one sample of cells taken before adoptive transfer (Fig. 4F), suggesting that gene delivery via pMHC-targeted viruses should not limit in vivo persistence of transduced cells relative to untransduced cells. In addition, tethered mIL-12 expression on tumor-infiltrating, transduced CD8 T cells was confirmed in mice receiving A1–mIL-12–transduced cells via flow cytometry (Fig. 4G), and a significant increase in the frequency of tumor-infiltrating CD8 T cells detected of total live cells was observed for these mice (fig. S6A). At the time tumors were harvested, there were no significant differences in tumor size (fig. S6B). Together, these results demonstrate that A1–mIL-12 transduced cells are able to survive and traffic to tumors in vivo where they mediate prolonged survival in an aggressive, syngeneic tumor model.

### Intravenously delivered pMHC virus targets naïve CD8 T cells in vivo

**Fig. 5.**
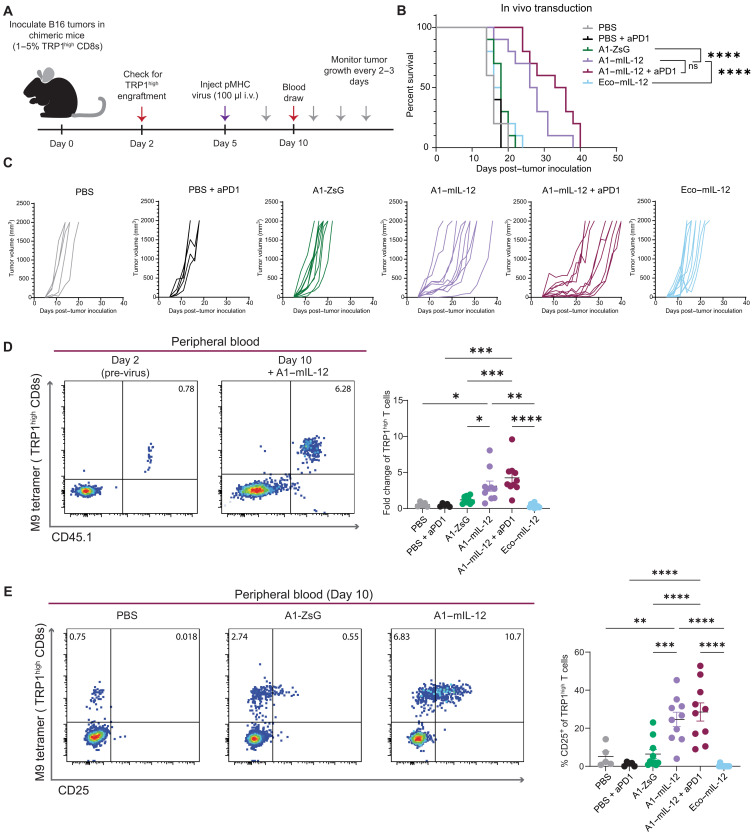
pMHC-targeted viruses delivering mIL-12 generate in vivo engineered antitumor T cells and extend survival in a syngeneic, solid tumor model. (**A**) B6 mice were irradiated and partially reconstituted with naïve TRP1^high^ lymphocytes. Two days later, 500,000 B16F10 tumor cells were inoculated subcutaneously. Five days after tumor inoculation, 100 μl of targeted viruses were injected intravenously (i.v.) via the tail vein. See table S1 for virus doses represented as total nanograms of p30 delivered. Gray arrows indicate intraperitoneal injections of anti-PD1 antibody (150 μg) on days 8, 11, 14, and 17. Red arrows indicate blood draws to evaluate TRP1^high^ T cell expansion. (**B**) Kaplan-Meier curve for overall survival. Representative of two independent experiments. *P* values were determined by a Bonferroni-corrected log-rank (Mantel-Cox) test. ****P < 0.0001 (**C**) Individual tumor growth curves from mice in (B). (**D**) Expansion of TRP1^high^ T cells calculated on the basis of the percentage of tetramer^+^ and CD45.1^+^ cells in the peripheral blood, where CD45.1 is a marker for transferred TRP1^high^ lymphocytes. Each flow cytometry plot is representative of one mouse and is gated on live CD8 T cells. Annotated percentages are of total, live CD8 T cells. Individual values with means ± SEM plotted. *P* values were calculated by Bonferroni-corrected one-way ANOVA. *P < 0.05, ***P < 0.001. (**E**) Expression of CD25 in peripheral blood samples from day 10, gated on live CD8 T cells. Each plot is representative of one mouse, and annotated percentages are of total, live CD8 T cells. Individual values with means ± SEM shown. *P* values were calculated via Bonferroni-corrected one-way ANOVA. **P < 0.01, ****P < 0.0001.

Considering the specificity of A1-targeted viruses in polyclonal populations in vitro, we investigated whether pMHC-targeted viruses could target antitumor T cells directly in vivo. B6 mice partially reconstituted with TRP1^high^ T cells were treated with A1- or Eco-targeted virus intravenously (Fig. 5A). A1–mIL-12 monotherapy mice or those receiving A1–mIL-12 in combination with anti-PD1 antibody (aPD1) experienced a significant extension in overall survival compared to mice treated with A1-ZsGreen or Eco–mIL-12 virus, underscoring that specific delivery of the therapeutic cargo to tumor-specific T cells was critical for therapeutic benefit (Fig. 5, B and C). Despite a modest increase in survival for A1–mIL-12 + aPD1 compared to A1–mIL-12, we observed no significant difference between these groups, suggesting that PD1 was not primarily responsible for the eventual loss of immune-mediated control.

Comparing TRP1^high^ T cell frequencies in the peripheral blood before and after virus injection, we saw the greatest fold expansion of TRP1^high^ T cells following injection of A1–mIL-12 virus compared to A1-ZsG, Eco–mIL-12, and the phosphate-buffered saline (PBS) control (Fig. 5D). TRP1^high^ CD8 T cells were specifically activated, as evidenced by an increase in CD25 expression post–virus injection (Fig. 5E), and although transduction was below the limit of detection for all groups, these results suggest that expansion of antigen-specific cells by pMHC displayed on the surface of viral particles amplifies the effect of a smaller number of cargo-expressing T cells. TRP1^high^ T cells exposed to A1-ZsG virus did not show a significant increase in CD25 expression compared to PBS controls, indicating that pMHC-targeted viruses can further fine-tune cell phenotype via delivery of a therapeutic cargo, as demonstrated here with tethered IL-12.

**Fig. 6.**
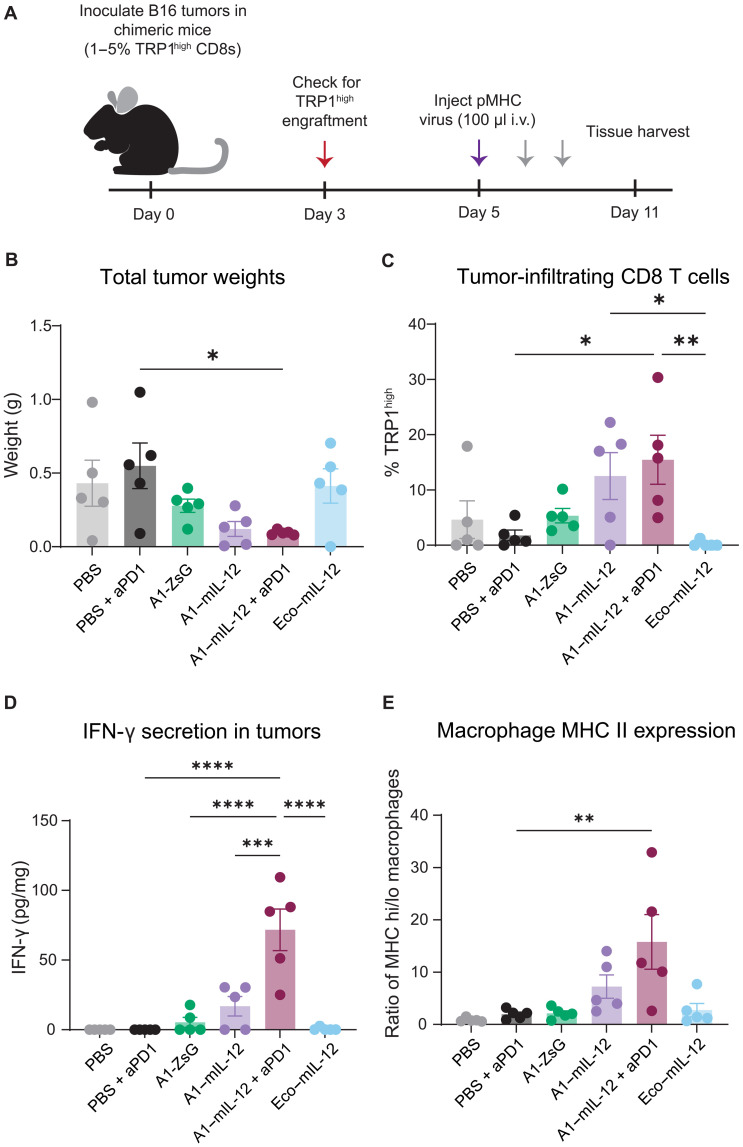
A1–mIL-12 viruses recruit adaptive and innate immune cells in an antitumor response. (**A**) B16F10 tumor cells (500,000) were inoculated subcutaneously on the left flank of chimeric C57BL/6J mice reconstituted with naïve TRP1^high^ T cells, comprising 1 to 5% of their total CD8^+^ population. Three days later, 100 μl of targeted virus was injected intravenously via the tail vein. See table S1 for virus doses in total nanograms of p30. The red arrow indicates a blood draw. Gray arrows indicate intraperitoneal injections of anti-PD1 antibody (150 μg) on days 7 and 10. On day 11, tissues were harvested for analysis via flow cytometry. (**B**) Tumor weights on day 11. Individual values plotted with means ± SEM. *P* values were calculated using Bonferroni-corrected one-way ANOVA. *P < 0.05. (**C**) Tumors were processed for flow cytometry and tumor-infiltrating TRP1^high^ T cells were quantified as a percentage of total tumor-infiltrating CD8 T cells. Individual values shown with means ± SEM. Significance was calculated using a Bonferroni-corrected one-way ANOVA. *P < 0.05, **P < 0.01. (**D**) IFN-γ secretion was quantified in tumor lysates and normalized to total tumor weight. Individual values depicted with means ± SEM. *P* values were determined using a Bonferroni-corrected one-way ANOVA. ***P < 0.001, ****P < 0.0001. (**E**) MHC II expression on tumor-associated macrophages was determined via flow cytometry. The ratio of MHC II^high^ to MHC II^low^ macrophages (MHC hi/lo) was calculated as a ratio of counts of each cell type. Individual values plotted with means ± SEM. Significance was determined using a Bonferroni-corrected one-way ANOVA. **P < 0.01

To explore changes in the tumor microenvironment that could explain observed differences in overall survival, tissues from a second cohort of mice were analyzed 6 days after treatment with virus, when tumor sizes began to diverge (Fig. 6, A and B). After A1–IL-12 + aPD1 injection, tumor-infiltrating TRP1^high^ T cells significantly increased compared to injections of PBS + aPD1 and Eco–mIL-12 (Fig. 6C). In addition, significantly more IFN-γ was detected in A1–IL-12 + aPD1 tumor lysates, indicating that tethered IL-12 initiates the anticipated downstream signaling in vivo, especially when combined with aPD1 (Fig. 6D). One well-characterized impact of an IFN-γ response in innate immune cells is the up-regulation of MHC class II (MHC II) by macrophages (*51*, *52*). We found that the ratio of tumor-infiltrating MHC II high to MHC II low macrophages was skewed toward an MHC II high response in mice injected with A1–mIL-12 + aPD1 compared to PBS + aPD1 or A1-ZsG (Fig. 6E). Collectively, these results underscore that delivery of tethered mIL-12 via pMHC-targeted viruses affects both adaptive and innate tumor-infiltrating immune cells, driving expansion of on-target cells and initiating a broader IFN-γ–based immune response within the tumor microenvironment.

### CD8 T cells activated in vivo with A1–mIL-12 viruses can form long-term memory

One of the most promising aspects of engineered cell therapy is the potential to induce long-lasting memory with a single dose of treatment. To evaluate whether pMHC-pseudotyped viruses delivering mIL-12 could generate a more robust immune response, we increased the dose of viruses delivered systemically to B16F10 tumor–bearing mice partially reconstituted with TRP1^high^ T cells, using intravenous and intraperitoneal routes of administration to accommodate increased volume given that intraperitoneal injections have been demonstrated to have similar bioavailability to intravenous injections in preclinical studies (fig. S7A) (*53*). Similar to previous in vivo transduction experiments, we saw specific expansion of the TRP1^high^ T cell population following addition of virus (fig. S7, B and C, and fig. S9A). These results demonstrate that even at high systemic doses, pMHC-targeted viruses remain targeted to antigen-specific T cells. At the same time point, we detected a significant increase in serum IFN-γ when delivering mIL-12 to tumor-specific T cells compared to nonspecific delivery of IL-12 (fig. S7D). Serum IL-12 levels were comparable between mice dosed with A1–mIL-12 and Eco–mIL-12 although higher than in PBS-treated controls (fig. S7E), suggesting that either endogenous IL-12 is being produced or that some of the tethered IL-12 is released from the cell surface. At this dose of systemically delivered virus, mice receiving A1–mIL-12 virus showed robust control of tumor growth, with 40% of mice alive at 40 days (fig. S7F). We evaluated the four remaining mice in the A1–mIL-12 group at day 40 to assess the presence and persistence of transduced TRP1^high^ T cells in peripheral blood as well as in tumors (fig. S8A). In two of the four mice, the majority of CD8 T cells were TRP1^high^ T cells (fig. S8B), and a detectable fraction of those TRP1^high^ T cells were stably transduced and exhibited either central memory (CD44^+^CD62L^+^) or effector memory (CD44^+^CD62L^−^) phenotypes, similar to nontransduced, but still antigen experienced, tetramer^+^ cells at this same time point (fig. S8, C to E). These results emphasize that in vivo–engineered, antigen-specific T cells can persist to become long-lived T cells following a single intravenous injection of pMHC-targeted virus. Examination of liver and spleen at this time point showed minimal ZsGreen expression, further indicating a lack of off-target transduction (fig. S9, B to F). Together, these data suggest that pMHC-targeted viruses are an efficient means of delivering potent immunomodulatory cargos that can be localized to an antigen-specific T cell population of interest in vivo.

**Fig. 7.**
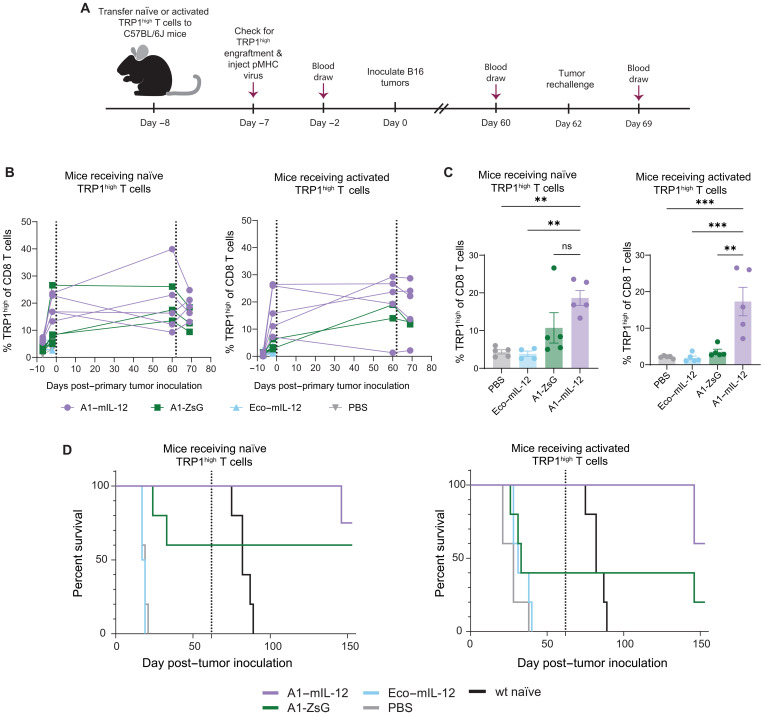
A single injection of pMHC-targeted virus is able to generate in vivo a robust TRP1^high^ T cell response that forms immunologic memory. (**A**) Freshly isolated TRP1^high^ CD8 T cells (naïve) (2.4 to 3 million) or 5 million TRP1^high^ CD8 T cells activated with anti-CD3/28 beads ex vivo for 48 hours (activated) were transferred into C57BL/6J mice. One day later, 100 μl of virus was injected intravenously. See table S1 for virus doses in total nanograms of p30. Seven days later, 500,000 B16F10 tumor cells were inoculated subcutaneously on the left flank. Peripheral blood samples were collected at time points indicated by the maroon arrows. At day 62, all surviving mice were rechallenged with the same dose of B16F10 tumor cells on the contralateral flank. (**B**) Frequencies of TRP1^high^ T cells (as a percentage of total CD8 T cells) in peripheral blood detected at the indicated time points in (A). Dotted lines indicate tumor inoculations. (**C**) Frequencies of TRP1^high^ T cells from (B) observed post–virus injection at day −2. Individual values plotted with means ± SEM. *P* values were calculated using a Bonferroni-corrected one-way ANOVA. ***P < 0.001, **P < 0.01. (**D**) Kaplan Meier curve for overall survival of mice in this study where *n* = 4 to 5 for all groups. Dotted line indicates secondary tumor challenge for all surviving mice. Simultaneously, a cohort of wild type C57BL/6J mice (naïve mice) were inoculated with tumors as controls to ensure expected tumor cell growth.

To further examine whether transduced cells could contribute to immunologic memory, we inoculated a new set of mice with TRP1^high^ CD8 T cells that were either naïve or previously activated in vitro with anti-CD3/CD28 beads for 2 days (Fig. 7A). Mice were then administered viruses by intravenous injection. Both A1-ZsG and A1–mIL-12 viruses robustly expanded TRP1-reactive CD8 T cells regardless of whether the CD8 T cells were naïve or recently activated (Fig. 7, B and C). This dramatic expansion was capable of controlling growth of subsequently inoculated B16 tumors. Mice receiving A1–mIL-12 viruses were protected from tumor growth with *n* = 10 of 10 mice surviving tumor-free. A1-ZsG virus treated mice were also capable of clearing B16 melanoma although not to the same extent (*n* = 5 of 10 tumor-free) (Fig. 7D). We then allowed the cured mice to rest to form a memory T cell pool. Unexpectedly, we observed high rates of TRP1^high^ T cell persistence, with adoptively transferred cells constituting 1.3 to 40% of total CD8 T cells in peripheral blood (Fig. 7B). Rechallenge with B16 tumors showed protective immunologic memory with all mice surviving longer than the tumor-naïve controls, a cohort of naïve B6 mice inoculated with the same tumor cell line, included to demonstrate the viability of injected tumor cells. Three mice in each cohort receiving A1–mIL-12 virus remained tumor free at the end of study (Fig. 7D). These results emphasize that antigen-specific T cells can persist to become long-lived memory cells following a single intravenous injection of pMHC-targeted virus. Together, these results underscore that pMHC-targeted viruses can be used to directly transduce antigen-specific T cells in vivo with a function-enhancing cargo, generating long-lived cells that orchestrate a robust antitumor immune response.

## DISCUSSION

The clinical success of TIL therapy and checkpoint blockade have underscored that preexisting antitumor T cells present in patient T cell repertoires can mediate robust clinical responses. To fully leverage the potential of these endogenous, tumor-specific T cells, we demonstrate that pMHC-targeted retroviruses can be engineered to target antigen-specific CD8 T cells for in vivo delivery of function-enhancing genetic cargos to modify T cell phenotype.

Our optimized pMHC pseudotyping strategy for gammaretroviruses facilitates efficient transduction, activation, and expansion of primary antitumor CD8 T cells. In TIL therapy, enriching for tumor-reactive T cells and minimizing bystander T cell involvement has been demonstrated to increase clinical efficacy (*54*). These data suggest that the ability of pMHC-targeted viruses to expand antigen-specific cells during the transduction process is a feature that could be important for the improved therapeutic outcomes we observe in both the ex vivo manufacturing and in vivo transduction models.

A major bottleneck in the development of cell therapy products is scaling the time-consuming and labor-intensive manufacturing process to meet growing clinical need. Because of these specialized infrastructure and skilled operator requirements, only a limited number of institutions are capable of manufacturing engineered cell therapies (*55*), further restricting patient access while increasing costs. Allogeneic cell products present one alternative to autologous cell engineering by providing off-the-shelf solutions. However, manufacturing allogeneic cell therapies still requires costly ex vivo manufacturing protocols similar to autologous cell therapies with the additional risk of generating alloreactive T cell responses. The enhanced specificity of pMHC-targeted viral vectors could bridge this gap by enabling in vivo engineering of autologous T cells. In the process of clinical translation, additional quality control measures to evaluate viral surface protein composition, particle aggregation (*56*), functional titer, and genomic copy number (*57*) will be necessary to ensure that the inherently heterogenous virus product meets safety and potency standards. We validate that pMHC-targeted viruses injected systemically are able to specifically expand and transduce tumor-specific T cells at low initial frequencies in vivo with minimal off-target transduction.

Although we observe transduced T cells in the tumor, antigen-specific T cell encounter with virus likely occurs systemically, with tumor-reactive T cells trafficking to the tumor microenvironment after transduction. Human patients with cancer responding to PD1 blockade have tumor-reactive T cells in circulation, as demonstrated by TCR clonotype tracking studies that have matched activated CD8 T cells from peripheral blood to clonotypes found in the tumor (*58*–*60*). We therefore predict that tumor-reactive T cells transduced in circulation would be able to traffic to the tumor, potentially contributing to clinical benefit in humans.

In this work, we demonstrate delivery of an immunomodulatory cargo that catalyzes improved outcomes in tumor-bearing mice. We chose a tethered IL-12 construct as a test cargo for our studies based on its narrow therapeutic index despite its ability to recruit multiple immune cell subtypes and orchestrate a favorable antitumor response. Prior work demonstrates the efficacy but dose-limiting toxicity of TILs engineered to secrete IL-12 (*28*). For this reason, efforts have been made to limit delivery of IL-12 to the tumor microenvironment (*50*, *61*), to require a hypoxic microenvironment to induce IL-12 secretion (*62*), or to directly tether the cytokine to endogenous or CAR-T cells (*63*–*65*). We report that our antigen-specific virus could be an effective way to safely dose mIL-12 systemically as only antitumor T cells are transduced with a spatially constrained construct.

While we expect that a wide variety of therapeutic proteins could be delivered using pMHC-targeted viruses, the specificity of these viruses uniquely enables delivery of potent immunomodulatory cargos. When delivering the proinflammatory cargo IL-12, we detected an increase in serum IFN-γ specifically in mice receiving A1–mIL-12 virus, an anticipated downstream molecule from IL-12 signaling in T cells. We expect that IFN-γ production and potential toxicities would be correlated with the starting frequency of antigen-specific T cells, similar to the precedent set by CAR-T cell therapy, where cytokine release syndrome is observed in patients with high initial tumor burden. Similar strategies to those implemented in engineered cell therapy could also be deployed to block inflammatory cytokines and control initial treatment-related adverse effects (*66*). To maximize therapeutic efficacy, future work could continue to explore whether certain cargos are especially synergistic with our pMHC viral pseudotyping strategy that also activates and expands target T cells.

The modularity and generality of our vectors could be leveraged for the simultaneous delivery of cargos to T cells recognizing a pool of antigens, further enhancing TIL therapy and reducing the potential for immune escape. A considerable challenge in implementing TIL therapy across broad patient populations is the limited number of shared neoantigen targets that are recognized by TILs (*67*, *68*). To increase patient compatibility, we envision targeting pMHC-displaying vectors with tumor-associated antigens such as MART-1 in melanoma (*69*) or HPV-derived antigens in cervical and oropharyngeal cancers (*70*). For a completely personalized approach, the pMHC-targeted vectors described here could be readily tailored to match an individual’s expressed antigens given that expression of single-chain trimers for viral targeting should be readily translated to all MHC alleles and can be incorporated into viral manufacturing without any additional steps beyond the inclusion of a gene encoding the single-chain pMHC. Tools to determine which pMHCs are recognized by tumor-specific TCRs already exist and could be leveraged to identify pMHCs that should be displayed as targeting molecules on the viral surface (*24*, *71*–*74*). Considering that antitumor T cells are often found at low frequencies in patient samples, we anticipate that our technology would pair well with therapeutic cancer vaccines containing one or a few known antigens, such as the KRAS or neoantigen vaccines currently in trials (*75*, *76*).

Although this demonstration of pMHC-targeted viruses was conducted within the setting of cancer immunotherapy, both the targeting and delivery components of our viral vectors are highly modular, making antigen-specific gene therapy using pMHC-targeted viral vectors a promising tool for development of cell therapies for a range of different diseases. Together, the pMHC-targeted vectors for gene delivery explored here could be powerful tools for improving the precision and quality of future cell therapy products.

## MATERIALS AND METHODS

### Study design

The objective of this study was to evaluate pMHC-targeted viruses as gene delivery vectors in an immunocompetent model system. We first validated that pMHC-targeted viruses can specifically modify different antigen-specific CD8 T cell populations by generating pMHC-targeted gammaretroviruses and isolating transgenic CD8 T cells for in vitro transduction. Subsequently, we adoptively transferred these ex vivo–engineered cells into mice bearing B16F10 tumors and monitored overall survival to determine whether therapeutic efficacy could be achieved following antigen-specific delivery of a function-enhancing cargo to tumor-specific T cells. Following an observed increase in overall survival for mice receiving A1–mIL-12–transduced T cells, we harvested tissues for flow cytometry analysis to verify persistence of engineered T cells. Last, we demonstrate that pMHC-targeted retroviruses can be injected systemically for in vivo engineering of tumor-specific T cells present at a starting frequency of 1 to 5% of the total CD8 T cell repertoire. With this model, we measured overall survival, evaluated tumor-infiltrating cells via flow cytometry, and quantified cytokine secretion in tumors and serum.

### Plasmids

The plasmid pCL-Eco was a gift from I. Verma (Addgene, plasmid # 12371; http://n2t.net/addgene:12371; RRID:Addgene_12371). The plasmid pUMVC was a gift from B. Weinberg (Addgene, plasmid # 8449; http://n2t.net/addgene:8449; RRID:Addgene_8449). MSCV ZsGreen plasmid was cloned by inserting an internal ribosomal entry site (IRES) followed by the coding region of ZsGreen into the MSCV vector backbone. pMD2.G was a gift from D. Trono (Addgene, plasmid # 12259; http://n2t.net/addgene:12259; RRID:Addgene_12259). The plasmid pMD2-VSVGmut was previously published and developed by our laboratory (Addgene, plasmid # 182229; http://n2t.net/addgene:182229; RRID:Addgene_182229) (*24*). To generate MSCV IL-12, mIL-12 was expressed as a tethered construct as previously described (*50*), consisting of the coding sequence of a single-chain mIL-12 (a gift from D. Wittrup) followed by a flexible G4S linker and residues 247 to 268 of the transmembrane domain of murine CD80 (UniProt ID Q00609). This tethered mIL-12 construct was inserted into the MSCV ZsGreen plasmid directly upstream of IRES-ZsGreen via digestion of the vector with XhoI/EcoRI (NEB) followed by Gibson Assembly. Correct assembly was validated via Sanger and Oxford Nanopore sequencing.

### Generation of HEK producer cell lines

For each pMHC of interest, we generated human embryonic kidney (HEK) 293T producer cell lines for stable mammalian cell expression of the targeting pMHC construct. To accomplish this, we cloned each single-chain pMHC into the pHIV vector and generated VSVG pseudotyped lentiviruses delivering a pMHC of interest. Lentiviruses were produced by transfecting 80 to 95% confluent HEK293T cells with pHIV-pMHC, psPAX2, and pMD2.G (encoding VSVG) plasmids at a mass ratio of 5.6:3:1. Plasmids were complexed with TransIT-Lenti Transfection Reagent (Mirus Bio) at a DNA mass ratio of 3:1 in OptiMEM (Thermo Fisher Scientific). After a 10-min incubation, DNA + TransIT-Lenti complexes were added dropwise to HEK293T cells. Forty-eight hours later, HEK293T supernatant was collected and filtered using a 0.45-μm low protein–binding filter, and unconcentrated viral aliquots were stored at −80°C. These VSVG pseudotyped viruses and diethylaminoethyl-dextran (8 μg/ml; Sigma-Aldrich) were added to freshly split HEK293Ts. After 48 hours, transduction was assessed via flow cytometry by trypsonizing and staining the transduced HEK293T cells with antibodies specific for the pMHC of interest. A pure population of pMHC^high^ HEK293T cells was then sorted using a fluorescence-activated cell sorter (FACS, Sony M900). Following the sort, pMHC^high^ HEK293T cells were expanded and frozen down to form producer cell line stocks.

### pMHC retrovirus production

pMHC-expressing HEK293T producer cell lines were thawed and passaged a minimum of three times in complete Dulbecco’s modified Eagle’s medium (DMEM), composed of DMEM [American Type Culture Collection (ATCC)] supplemented with 10% fetal bovine serum and penicillin-streptomycin (100 U/ml; Corning). The cells were then cultured to 80 to 95% confluency and transfected with pCL-Eco and an MSCV transfer plasmid (MSCV-ZsGreen or MSCV-mIL-12) at a mass ratio of 3:5. Plasmids were complexed with TransIT-LT1 Transfection Reagent (Mirus Bio) at a mass ratio of 1:3 in OptiMEM (Thermo Fisher Scientific). After incubating for 25 min, DNA + TransIT-LT1 complexes were added dropwise to HEK293T cells. Supernatants were collected at 48 hours posttransfection and filtered with a 0.45-μm low protein–binding filter. The cell medium was replaced with fresh complete DMEM plus 25 mM Hepes, and a second collection was conducted at 72 hours posttransfection.

Unconcentrated virus was immediately frozen at −80°C for future assays. Concentrated virus was produced by pooling supernatants from 48- and 72-hour collections and adding a poly(ethylene glycol) (PEG) concentration cushion composed of 45% PEG, molecular weight 6000 in PBS at a volume of 1:3 PEG cushion to virus supernatant. Virus supernatant plus PEG cushion were stored at 4°C overnight and subsequently centrifuged at 1500*g* for 45 min. The supernatant from this spin was discarded, and the PEG + virus pellet was resuspended in OptiMEM (Thermo Fisher Scientific) at 1/100 of the volume of the harvested HEK supernatant. This concentrated virus was then aliquoted and stored at −80°C.

### Animals

C57BL/6J mice aged 6 to 8 weeks were purchased from the Jackson Laboratories. All animal studies were performed in accordance with guidelines approved by the MIT Division of Comparative Medicine as well as the MIT Committee on Animal Care (Institutional Animal Care and Use Committee, protocol number 0621-032-24) and in accordance with DFCI IACUC-approved protocols (14-019 and 14-037). TRP1^high^ TCR transgenic mice were bred in-house, and mice aged 8 to 24 weeks were used as T cell donors. 2C TCR and OT-I transgenic mice were gifts from the Spranger and Irvine laboratories, respectively, and bred in-house at the Koch Institute (MIT) mouse facility. Chimeric mice for in vivo pMHC-targeting were generated by irradiating C57BL/6J with 1 Gy. Four to six hours later, lymphocytes from TRP1^high^ mice were transferred intravenously via tail vein injection. All animal work was conducted in compliance with the National Health Institutes/National Cancer Institute ethical guidelines for tumor-bearing animals.

### Isolation and transduction of murine CD8 T cells

CD8 T cells were isolated from spleens and inguinal lymph nodes of transgenic and B6 mice, with each strain processed separately. Tissues were pooled and filtered through a 70-μm filter to create single-cell suspensions. Downstream processing was conducted following Easy Sep Mouse CD8+ T cell isolation kit (STEMCELL Technologies) or Miltenyi Biotec CD8a^+^ T Cell Isolation Kit for murine cells following the manufacturer’s instructions (Miltenyi Biotec). Isolated CD8 T cells were cultured at 1 million cells/ml in RPMI 1640 (ATCC) supplemented with 10% fetal bovine serum, penicillin-streptomycin (100 U/ml; Corning), 50 μM β-mercaptoethanol (Thermo Fisher Scientific), 1% sodium pyruvate (Thermo Fisher Scientific), 1% nonessential amino acids (Thermo Fisher Scientific), and mIL-2 (10 ng/ml; Sigma-Aldrich). To transduce cells, polybrene (8 μg/ml; Santa Cruz Biotechnology), and concentrated virus were added followed by spinfection (1000*g* at 32°C for 1.5 hours).

### Tumor inoculations for in vivo experiments

B16F10 cells were cultured until 80 to 90% confluent, then trypsonized, washed with PBS, and resuspended in Hanks’ balanced salt solution at 2 × 10^6^ cells/ml. Mice were shaved 1 day before inoculations, and 250 μl (5 × 10^5^) cells were injected subcutaneously into the left flank. Before injections, the mice were randomly assigned to treatment groups. Baseline weight was measured on injection day for injections of volumes less than or equal to 100 μl or 1 day after injections of larger volumes. Body weight was monitored two to three times every week. In survival experiments, tumor size was measured in three dimensions (length, width, and height) every 2 to 4 days. Mice were euthanized when tumor volume exceeded 2000 mm^3^, tumors showed evidence of ulceration, or weight decreased 20% relative to baseline.

### Ex vivo transduction for adoptive transfer experiments

CD8 T cells to be transduced by Eco-targeted gammaretroviruses were isolated 3 days before adoptive transfer and were activated with 10 μl/M cells using Dynabeads mouse T-activator CD3/CD28 beads (Thermo Fisher Scientific) for 24 hours. Two days before adoptive transfer, preactivated cells were debeaded, and all cells were transduced following the above in vitro protocol. Equivalent numbers of preactivated or freshly isolated T cells were transduced across all groups. Virus doses were standardized across groups so that the same nanograms of p30/M cells was added in every condition, where p30 ng/ml for each virus was determined by MuLV Core Antigen ELISA (Cell Biolabs). Across all three replicate experiments, a dose of 7.9 × 10^3^ to 1.2 × 10^4^ ng of p30/M cells was used to transduce CD8 T cells. For cells transduced by A1-targeted viruses, no preactivation was required, and InVivoMAb anti-CD28 antibody (5 μg/ml; BioXCell) was added in conjunction with concentrated virus before spinfection. On transfer day, the cells were counted and stained to assess viability, activation, and percent transduction. All cells in the resultant cell product for each group were transferred, with a minimum of 800,000 transduced cells injected per mouse and not exceeding a maximum dose of 10.3 M total cells per mouse.

### In vivo transduction experiments

B16F10 tumors were inoculated in chimeric C57BL/6J mice as described above. Where indicated, baseline frequencies of TRP1^high^ CD8 T cells in peripheral blood was quantified via a retro-orbital bleed. Depending on the study, 100 to 300 μl of virus was injected intravenously into the tail vein with an additional 200 μl injected intraperitoneally in studies where 500 μl total virus was delivered. In experiments where a combination of virus plus checkpoint blockade was tested, anti-PD1 (clone RMP1-14, BioXCell) was administered at a 150 μg dose in endotoxin-free PBS delivered intraperitoneally at the time points specified for a maximum of four total doses.

### Tissue collection for histology

Livers and spleens were collected and placed in fixative, Z-fix (Thermo Fisher Scientific, NC9378601), for 30 to 45 min and then left in 30% sucrose in PBS overnight at 4°C. Tissues were embedded in OCT blocks and frozen by floating on liquid nitrogen before storage in −80°C. Sections were acquired using a Leica CM3050S Cryostat sectioned at −20°C at 7 μm per slide. Staining was performed by washing with 0.1% Triton-X 100 in PBS for 3 × 15 min and then staining for CD3–phycoerythrin (PE) (1:100, BioLegend, 100205) overnight at room temperature. Slides were washed in 0.1% Triton-X 100 in PBS for 3 × 15 min and then stained with 4′,6-diamidino-2-phenylindole (1:2000) for 30 min at room temperature in the dark. Slides were washed once in PBS before being mounted in Prolong Gold Antifade Mountant (Thermo Fisher Scientific, P36930) and imaged using an Olympus IX73 fluorescent microscope with a Hamamatsu Orca-Spark C11440-36 U camera.

### Antibodies and flow cytometry

For flow cytometry analysis, cells were first washed with PBS and then stained 1:500 with LIVE/DEAD Fixable Viability Dye (Thermo Fisher Scientific) and 1:200 with purified anti-mouse CD16/32 (BioLegend, clone 93) for 15 min on ice. If CD8 T cells were isolated, no purified anti-mouse CD16/32 was used. Following an additional wash step, this time in FACS buffer (PBS mixed with 0.5% bovine serum albumin and 2 mM EDTA), samples were stained with fluorescently labeled antibodies at 1:200 dilution from the stock solution. The cells were then washed once with FACS buffer before analysis on a Cytoflex S (Beckman) or Sony Biotechnology SP6800 Spectral Analyzer. For experiments where absolute cell counts were calculated, CountBright Absolute Counting Beads (Thermo Fisher Scientific) were added to each well just before analysis. M9 tetramers, displaying the TRP1 epitope TAPDNLGYM in the context of MHC H2-D^b^, were produced in-house: Single-chain pMHC monomers were expressed in High Five cells (Thermo Fisher Scientific), purified, and biotinylated. Monomers were then mixed with PE streptavidin (BioLegend) at a ratio of 5:1 in PBS and incubated on ice for 10 min. When relevant, tetramers were added to cells concurrently with antibodies, which were then collectively incubated for 30 min at room temperature.

Tumors were processed for flow cytometry by removing the entire tumor and using scissors to manually dissociate the tissue. Samples were then incubated in RPMI 1640 supplemented with tumor digestion enzymes (Miltenyi tumor dissociation kit, catalog no. 130-096-730) at 37°C for 30 min. Following digestion, the samples were filtered through a 40-μm strainer to ensure generation of single-cell suspensions. After harvest, spleens were processed by filtering through a 40-μm strainer and performing one round of ACK lysis (Thermo Fisher Scientific). Lymph nodes were ground through 40-μm filters and resuspended for downstream staining. Peripheral blood was subjected to two rounds of ACK lysis before subsequent staining.

### pSTAT4 intracellular staining

CD8 T cells from a TRP1^high^ mouse were isolated as described above and transduced with A1-ZsG or A1–mIL-12 viruses following the same in vitro transduction protocol outlined previously. Two days after transduction, the cells were fixed by adding an equal volume of prewarmed BD Phosflow Fix Buffer I (BD Biosciences) to the media and incubating for 10 min at 37°C. The cells were washed twice with FACS buffer and fixed with BD Phosflow Perm Buffer III for 30 min on ice. After permeabilization, the cells were washed twice with FACS buffer and stained with 647 anti-pSTAT4 (pY693) (1:50) for 1 hour on ice in the dark. Following staining, the cells were washed with FACS buffer and acquired on a Cytoflex S (Beckman).

### IFN-γ and IL-12 ELISAs

Enzyme-linked immunosorbent assay (ELISA) MAX Deluxe Set Mouse IFN-γ (BioLegend) and ELISA MAX Standard Set Mouse IL-12/IL-23 (p40) (BioLegend) were used to quantify IFN-γ production and untethered IL-12, respectively. Assays were conducted according to the manufacturer’s instructions. For ELISAs of cell supernatants from in vitro cultures, supernatants were collected 48 hours after transduction and frozen at −80C until the time of assay.

Tissue samples for IFN-γ analysis were collected 11 days post–tumor inoculation and flash frozen on the day of tissue harvest. To make tumor lysate, tumors were thawed in 100 to 300 μl radioimmunoprecipitation assay (RIPA) lysis buffer with a protease inhibitor (Millipore Sigma) and phosphatase inhibitor cocktail (Cell Signaling Technology) and transferred to bead beating tubes (OMNI). Tubes were placed in a Bead Ruptor, and tumors were mechanically dissociated for 30 s. Tumor lysate was then centrifuged at 13,000*g* for 20 min at 4°C. Supernatant was collected and frozen at −80C for subsequent processing. Pierce BCA Protein Assay kits (Thermo Fisher Scientific) were used to determine tumor lysate protein concentration for normalization.

### Software

Graphs were generated using GraphPad Prism 10. Flow cytometry data were analyzed by FlowJo (10.10.0). Figures were composed and compiled using Adobe Illustrator.

### Statistical analysis

GraphPad Prism 10 was used to conduct statistical analyses. Results were considered significant if *P* < 0.05. *P < 0.05; **P < 0.01; ***P < 0.001; ****P < 0.0001.
