# Supplementary material for: Peptide-MHC–targeted retroviruses enable in vivo expansion and gene delivery to tumor-specific T cells
Source: Sci Adv. 2025 Nov 7;11(45):eadv2331. doi: 10.1126/sciadv.adv2331 (PMC12594172; doi:10.1126/sciadv.adv2331)
Supplement: Supplementary file 1 — Figs. S1 to S9 Table S1 [file sciadv.adv2331_sm.pdf]

Science Advances
AAAS

Supplementary Materials for

Peptide-MHC–targeted retroviruses enable in vivo expansion and gene delivery to tumor-specific T cells

Ellen J. K. Xu *et al.*

Corresponding author: Michael E. Birnbaum, mbirnb@mit.edu;
Stephanie K. Dougan, stephanie_dougan@dfci.harvard.edu

Sci. Adv. **11**, eadv2331 (2025)
DOI: 10.1126/sciadv.adv2331

This PDF file includes:

Figs. S1 to S9
Table S1

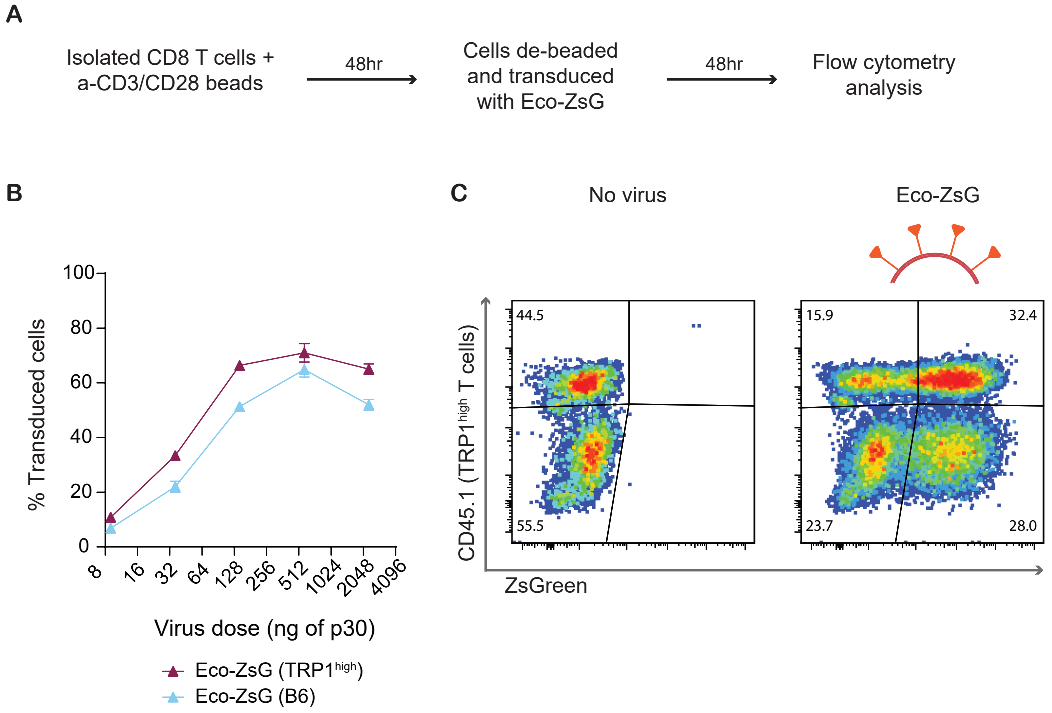

Fig. S1. Eco-ZsGreen viruses efficiently transduce activated CD8 T cells in an antigen-independent manner. (A) Experimental timeline evaluating Eco-ZsG transduction in activated cells. Isolated TRP1^{high} and B6 CD8 T cells were mixed at a 1:1 ratio prior to addition of beads. **(B)** Results from (A) 48 hours after addition of virus. Percent transduced cells was calculated as a fraction of total TRP1^{high} or B6 cells. Mean ± SEM plotted (n = 3). **(C)** Representative plots from data sumarized in (B) demonstrating ZsGreen expression observed with addition of Eco-ZsG (right) compared to the no virus condition (left).

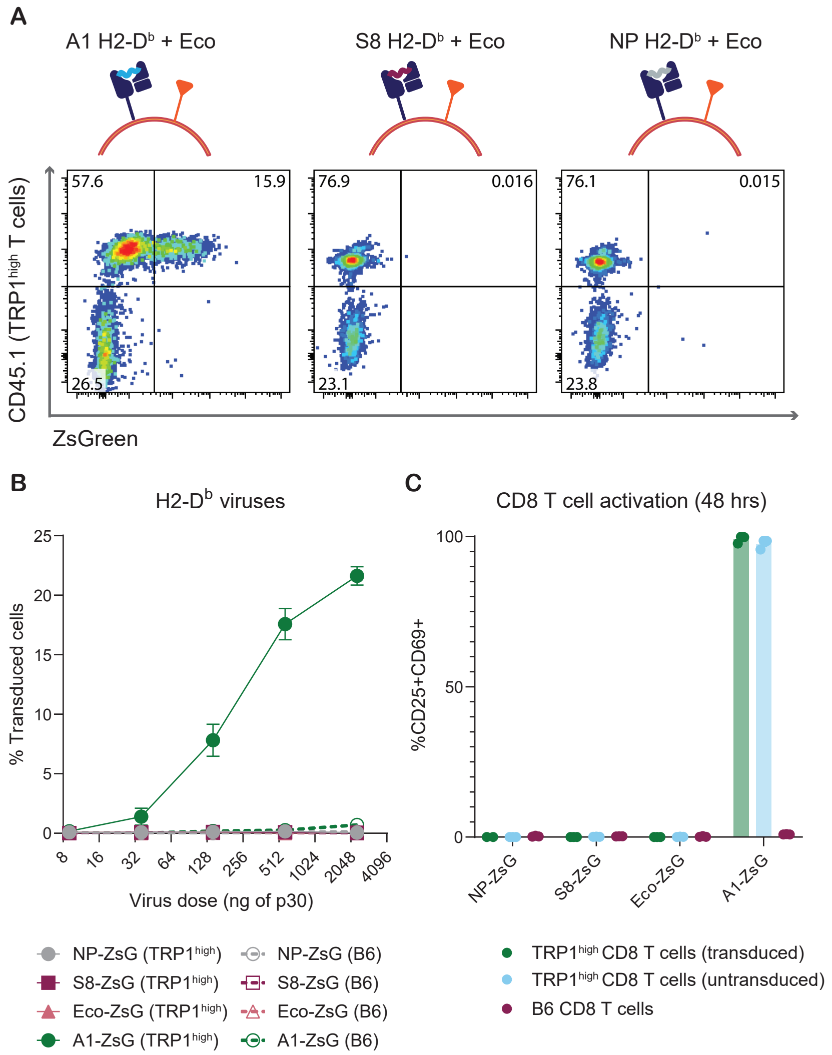

Fig. S2. pMHC-targeted viruses presenting off-target peptides in H2-D^b complexes do not mediate transduction of TRP1^{high} CD8 T cells. (A) pMHC-targeted viruses, each displaying a different peptide presented by H2-D^b, were added to transduce a mixture of on-target (TRP1^{high}) and off-target (B6) CD8 T cells. Transduction was assayed after 48 hours with representative flow plots shown. **(B)** Summary data from (A) where percent transduced was calculated as a fraction of the total on-target or off-target population. Values are mean ± SEM. **(C)** Summary plot showing percent CD25+CD69+ after addition of different pMHC-targeted viruses (2310ng of p30 dose). TRP1^{high} transduced, TRP1^{high} untransduced, and B6 populations correspond to the same populations defined in Fig. 1D. Individual values with mean ± SEM shown.

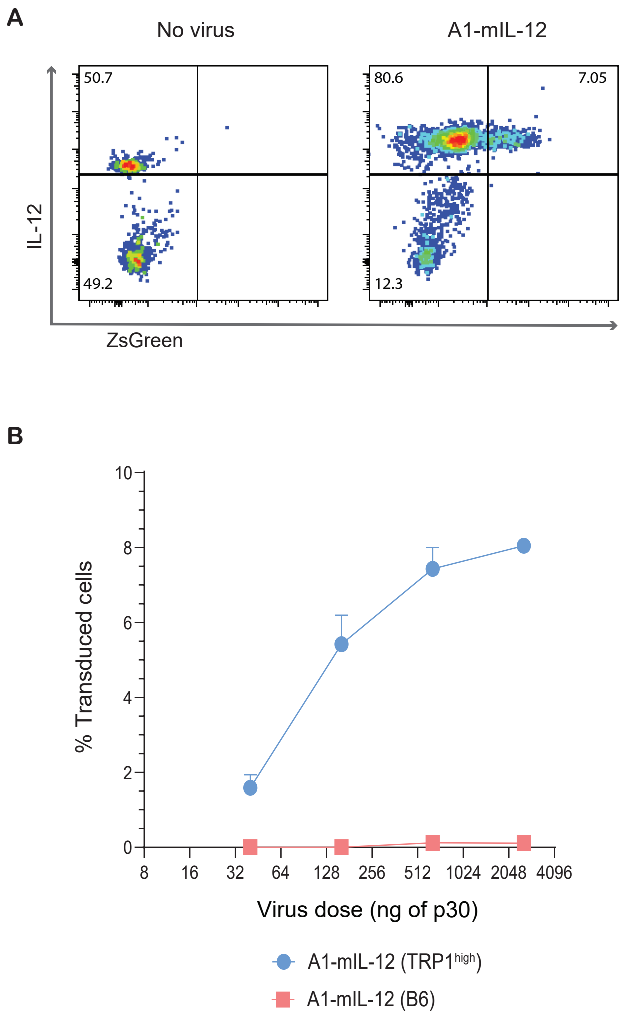

Fig. S3. A1-targeted viruses retain their ability to transduce TRP1^{high} T cells specifically when delivering a function-enhancing cargo, mIL-12. (A) Representative flow plots of transduction achieved after adding A1-mIL-12 virus at a dose of 2576ng of p30 (right) to a 1:1 mixture of TRP1^{high} and B6 CD8 T cells measured after 48 hours. Single experiment from n = 3 biological replicates. **(B)** Summary plot of (A) where n = 3 technical replicates. Percent transduced cells was calculated as a fraction of the total on-target or off-target populations with mean ± SEM shown.

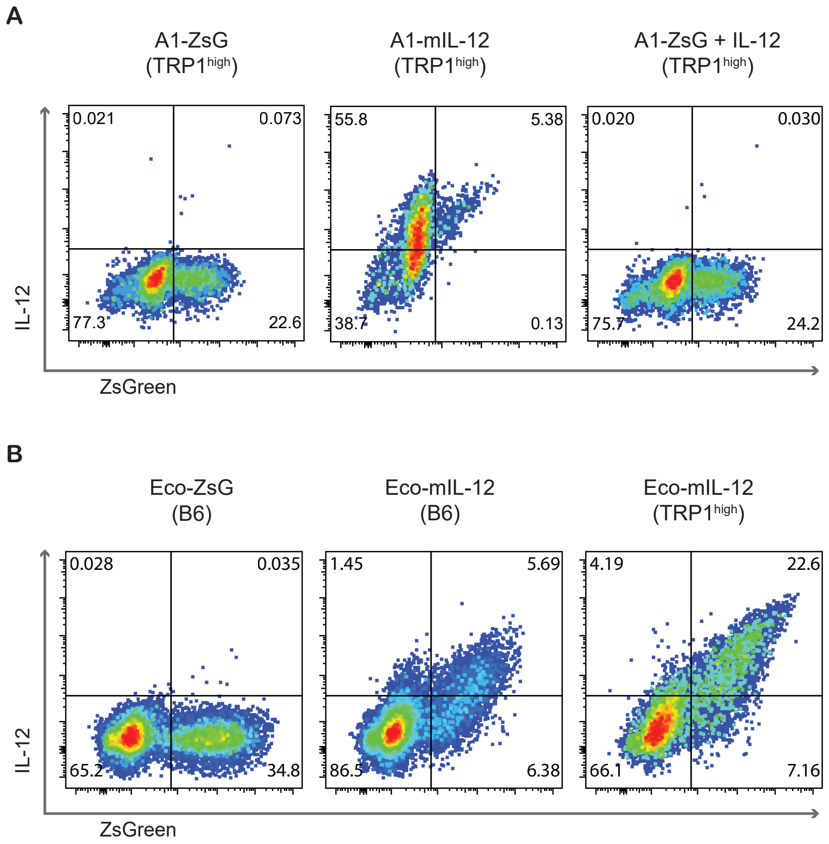

Fig. S4. Tethered IL-12 is expressed in conditions where secreted IFN-γ is detected in the supernatant. (A) Representative flow plots of the TRP1^{high} T cells transduced in the experiment shown in Fig. 3C. In this experiment, both B6 and TRP1^{high} CD8 T cells were isolated and transduced with A1-targeted viruses. Two days later, cells were analyzed via flow cytometry for IL-12 and ZsGreen expression at the same time that supernatants were collected for IFN-γ ELISA. **(B)** Representative flow plots of the T cells transduced in the experiment shown in Fig. 3D. B6 and TRP1^{high} CD8 T cells were activated with anti-CD3/28 beads. After two days, cells were de-beaded and transduced with Eco-targeted viruses. Two days after transduction, cells were analyzed via flow cytometry and supernatants were collected for IFN-γ ELISA.

A

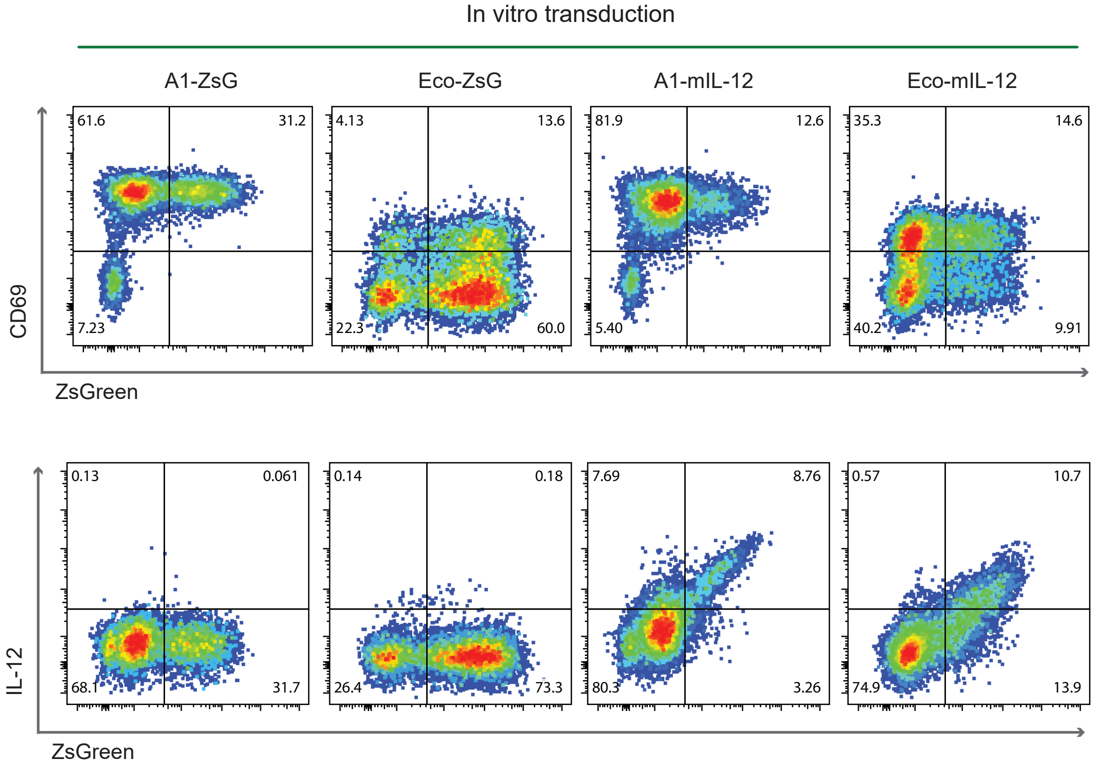

B

Virus	Cells	Total cells transferred/mouse	Transduced cells transferred/mouse
A1-ZsG	TRP1^{high}	10.3M	3.2M
Eco-ZsG	TRP1^{high} (preactivated)	10.3M	7.6M
A1-mIL-12	TRP1^{high}	10.3M	1.3M
Eco-mIL-12	B6 (preactivated)	10.3M	2.5M

Fig. S5. TRP1^{high} T cells are transduced and activated during ex vivo production process prior to adoptive transfer. (A) Evaluation of transduction, as a measure of ZsGreen expression, activation, as a measure of CD69 expression, and tethered IL-12 expression of each group of *in vitro* transduced cells two days after addition of virus, preceding adoptive transfer. **(B)** Table outlining the cells (TRP1^{high} or B6, preactivated or freshly isolated) that were transduced in each group. A total of 10.3M cells were transferred per mouse in each treatment group, but due to inherent differences in the number of transducing units per mL of each virus, different quantities of total transduced cells per mouse were transferred.

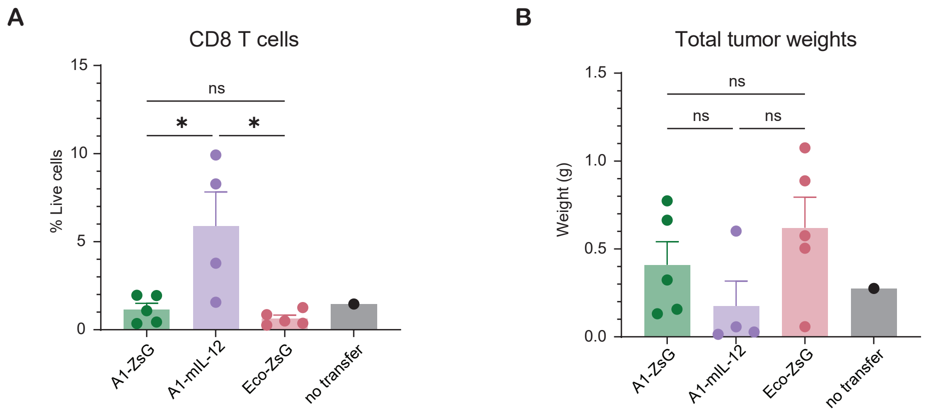

Fig. S6. Mice treated with A1-mIL-12 transduced TRP1^high T cells have a higher proportion of CD8 T cells and no significant differences in total tumor size. (A) Frequency of CD8 T cells out of total live cells in tumors harvested 5 days after adoptive transfer. Individual values are plotted with mean ± SEM. P values were calculated using Bonferroni-corrected one-way ANOVA. * $p < 0.05$. **(B)** Tumor weights on day 10 post tumor inoculation, 5 days after transfer of *ex vivo* transduced cells. Individual values are plotted with mean ± SEM. P values were calculated using Bonferroni-corrected one-way ANOVA. * $p < 0.05$.

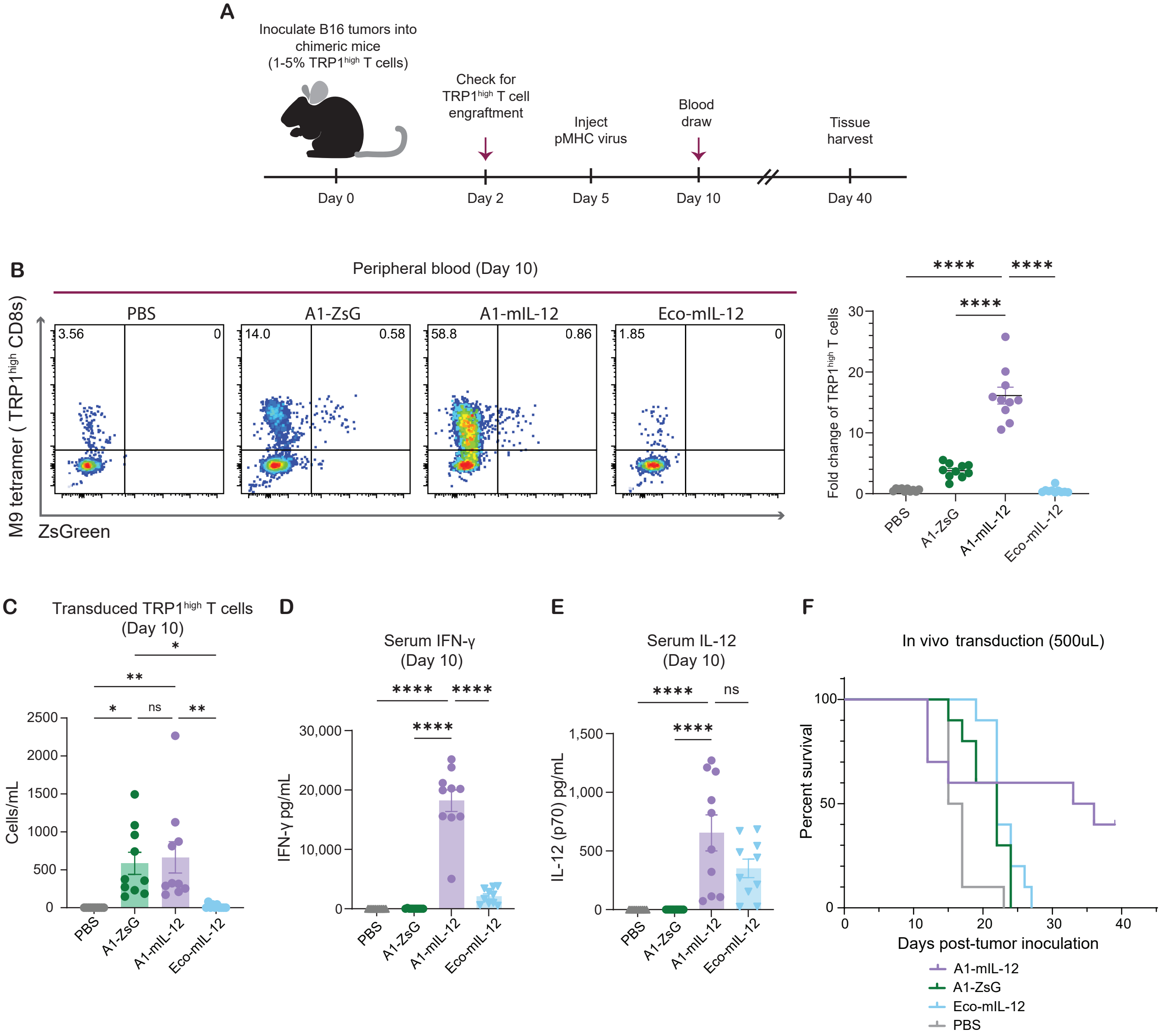

Fig. S7. Increasing A1-mIL-12 dose amplifies TRP1^high expansion and transduction in vivo. (A) 500,000 B16F10 tumor cells were inoculated subcutaneously on the left flank of chimeric C57BL/6J mice reconstituted with naïve TRP1^high T cells. Five days later, a total of 500uL of virus was injected (300uL i.v. + 200uL i.p.). See table S1. for virus doses in total ng of p30. Maroon arrows indicate blood draws. **(B)** Representative plots from peripheral blood draw on day 10 from one mouse in each group. Annotated percentages are of total, live CD8 T cells. Fold change of TRP1^high T cells was calculated by comparing the frequency of TRP1^high T cells at day 10 with their initial frequency at day 2. Individual values depicted with mean ± SEM. P values were calculated using a Bonferroni-corrected one-way ANOVA. **** p < 0.0001 **(C)** Absolute counts of transduced TRP1^high T cells detected at day 10 in peripheral blood. Individual values shown with mean ± SEM. P values were determined using a Bonferroni-corrected one-way ANOVA. * p < 0.05, **p < 0.01 **(D + E)** On day 10, serum cytokine concentration was determined by IFN-γ ELISA (D) or IL-12 (p70) ELISA (E). Individual values depicted with mean ± SEM. P values were assigned using a Bonferroni-corrected one-way ANOVA. **** p < 0. **(F)** Kaplan Meier curve for overall survival of mice in this study. Representative of two different experiments where n = 10 mice per group.

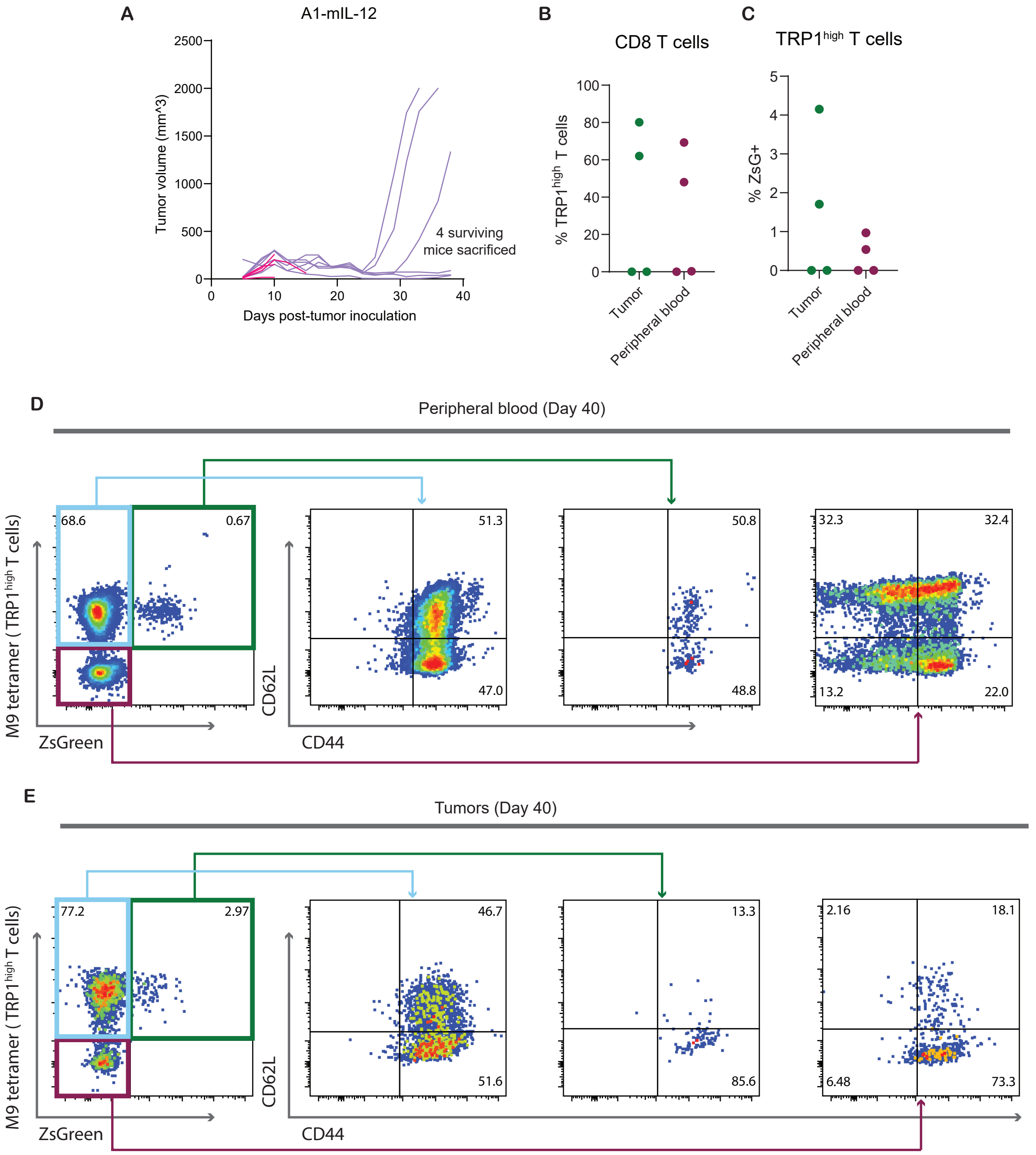

Fig. S8. A1-mIL-12 virus is able to generate *in vivo* memory TRP1^{high} CD8 T cells in a subset of treated mice. (A) Tumor volume measurements from the A1-mIL-12 treated group. Four of these mice were euthanized at early timepoints upon fulfillment of humane endpoint criteria due to initial treatment toxicities (shown in pink). Two mice were euthanized when tumor size reached maximum volume, and four mice maintained relatively small tumors until day 40 when all remaining animals were sacrificed to determine persistence and trafficking of transduced TRP1^{high} T cells. **(B)** Frequencies of TRP1^{high} T cells in tumor and peripheral blood detected in two of the four total mice at day 40. **(C)** Frequencies of transduced TRP1^{high} T cells in TRP1^{high} populations detected in (B). **(D-E)** Representative plots of memory phenotypes of circulating (D) or tumor-infiltrating (E) T cells. Naïve T cells = CD62L+CD44-, central memory T cells = CD62L+CD44+, effector memory T cells = CD62L-CD44+, stem cell-like memory = CD62L-CD44-. The first flow panel is gated on live CD8 T cells, with subsequent panels gated on untransduced TRP1^{high} T cells, transduced TRP1^{high} T cells, and tetramer negative CD8 T cells as indicated by the boxes and arrows.

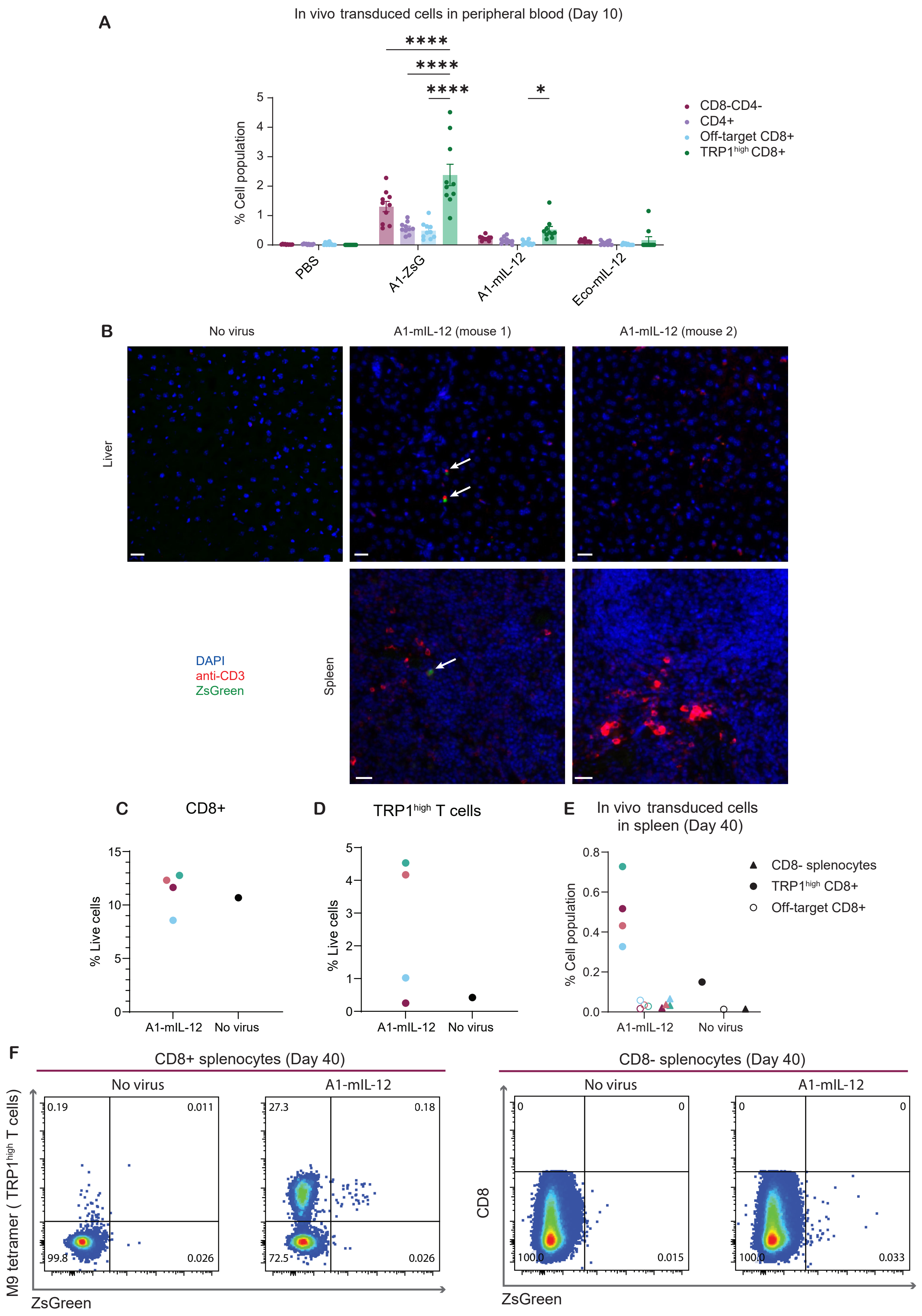
A
In vivo transduced cells in peripheral blood (Day 10)
% Cell population
PBS
A1-ZsG
A1-mIL-12
Eco-mIL-12
CD8-CD4-
CD4+
Off-target CD8+
TRP1high CD8+
B
No virus
A1-mIL-12 (mouse 1)
A1-mIL-12 (mouse 2)
Liver
Spleen
DAPI
anti-CD3
ZsGreen
C
CD8+
% Live cells
A1-mIL-12
No virus
D
TRP1high T cells
% Live cells
A1-mIL-12
No virus
E
In vivo transduced cells in spleen (Day 40)
% Cell population
CD8- splenocytes
TRP1high CD8+
Off-target CD8+
A1-mIL-12
No virus
F
CD8+ splenocytes (Day 40)
No virus
A1-mIL-12
M9 tetramer (TRP1high T cells)
ZsGreen
CD8- splenocytes (Day 40)
No virus
A1-mIL-12
CD8
ZsGreen

Fig. S9. At high systemic doses, pMHC-displaying virus remains largely targeted towards on-target cells with minimal off-target transduction detected in livers and spleens of mice treated with A1-mIL-12 virus. (A) ZsGreen+ cells detected in peripheral blood at day 10, 5 days after injection of 500uL of virus. Transduction is quantified as a frequency of the total cell population, defined by the indicated markers. Individual values with mean ± SEM plotted. P values were determined by Bonferroni-corrected two-way ANOVA. * $p < 0.05$, **** $p < 0.0001$ **(B)** Tissues from survivng mice were harvested at day 40, fixed, and sectioned for subsequent analysis. Shown are representative images from an untreated mouse and two mice treated with A1-mIL-12, where each image is from a different mouse. Tissues were stained with DAPI (blue) and anti-CD3 (red) alongside ZsGreen (green). Images shown are from livers (top row) and spleens (bottom row). White arrows highlight instances of ZsGreen. Selected images were chosen to maximize display of detectable ZsGreen in the tissue of interest. Scale bars shown in white are 20um. **(C-E)** Spleens from mice harvested at Day 40 were analyzed via flow cytometry, where each mouse is assigned a unique color. **(F)** Representative results from data summarized in (E).

Table S1. Virus doses for in vivo transduction experiments.

	total volume (uL)	total ng of p30
**Figure 5**		
A1-ZsG	100	7.49E+03
A1-mIL-12	100	3.94E+04
A1-mIL-12 + a-PD1	100	3.94E+04
Eco-mIL-12	100	2.48E+04
**Figure 6**		
A1-ZsG	100	7.49E+03
A1-mIL-12	100	8.71E+03
A1-mIL-12 + a-PD1	100	8.71E+03
Eco-mIL-12	100	2.48E+04
**Figure S7**		
A1-ZsG	500	3.74E+04
A1-mIL-12	500	4.35E+04
Eco-mIL-12	500	1.24E+05
**Figure 7**		
A1-ZsG	100	1.79E+04
A1-mIL-12	100	3.94E+04
Eco-mIL-12	100	2.48E+04
